# Automated Defect and Correlation Length Analysis of Block Copolymer Thin Film Nanopatterns

**DOI:** 10.1371/journal.pone.0133088

**Published:** 2015-07-24

**Authors:** Jeffrey N. Murphy, Kenneth D. Harris, Jillian M. Buriak

**Affiliations:** 1 Department of Chemistry, University of Alberta, Edmonton, Alberta, Canada; 2 National Institute for Nanotechnology (NINT), Edmonton, Alberta, Canada; Universita' degli Studi del Salento, ITALY

## Abstract

Line patterns produced by lamellae- and cylinder-forming block copolymer (BCP) thin films are of widespread interest for their potential to enable nanoscale patterning over large areas. In order for such patterning methods to effectively integrate with current technologies, the resulting patterns need to have low defect densities, and be produced in a short timescale. To understand whether a given polymer or annealing method might potentially meet such challenges, it is necessary to examine the evolution of defects. Unfortunately, few tools are readily available to researchers, particularly those engaged in the synthesis and design of new polymeric systems with the potential for patterning, to measure defects in such line patterns. To this end, we present an image analysis tool, which we have developed and made available, to measure the characteristics of such patterns in an automated fashion. Additionally we apply the tool to six cylinder-forming polystyrene-*block*-poly(2-vinylpyridine) polymers thermally annealed to explore the relationship between the size of each polymer and measured characteristics including line period, line-width, defect density, line-edge roughness (LER), line-width roughness (LWR), and correlation length. Finally, we explore the line-edge roughness, line-width roughness, defect density, and correlation length as a function of the image area sampled to determine each in a more rigorous fashion.

## Introduction

The ability of block copolymers (BCPs) to self-assemble into periodic structures, with periods ranging from 5 nm to well over 100 nm, has prompted investigation into their potential applications for nanopatterning of integrated circuits,[1–4] bit-patterned storage media,[4–7] optical devices,[8,9] tissue interfacing,[10–12] and others. Lamellar or cylindrical domains of block copolymers can be used to create linear structures,[13,14] both large[8,15] and small,[16] when confined in one dimension as thin films on substrates with appropriate wetting characteristics.[17–19] Such patterns can be used as lithographic masks through etching or as scaffolds to create other nanostructured surfaces and materials.[20,21]

For application in semiconductor fabrication, the International Technology Roadmap for Semiconductors (ITRS) has, in its Directed Self-Assembly Critical Assessment (where the term directed self-assembly is represented by the acronym DSA), identified challenges in 15 metrics, including:[22] Feature sizes of under 10 nm, the ability to “add, exclude or trim individual DSA…features with simple lithography”,^22^ a low degree of line edge roughness (LER, 3σ) < 0.6 nm, defect density less than 0.01 cm^-2^, and an annealing time of less than one minute. In addition, surfaces require appropriate wetting characteristics and surface energies in order to enable the process of self-assembly in the desired orientation with respect to the surface plane. These metrics have been correctly identified as challenges as they are daunting goals, but they represent very clear, quantified metrics that need to be attained. Lacking, however, is a unified method of accurately determining each parameter ‘in the field’, with actual samples of surfaces patterned *via* block copolymer self-assembly (*vide infra*).

Much of the work to optimize BCP DSA has been carried out with a narrow range of polymers, namely polystyrene*-block*-poly(methyl methacrylate) (PS-*b*-PMMA),[1,23,24] polystyrene-*block*-polydimethylsiloxane (PS-*b*-PDMS),[2,25–27] and polystyrene-*block*-poly(2-vinylpyridine) (PS-*b*-P2VP).[10,14,28,29] Each of these polymers possesses favorable characteristics for nanopatterning, but many other block copolymer systems still remain to be designed, synthesized, and investigated, as the exploration of the space of possible systems, including structural classes and chemical motifs (monomers) is nowhere near complete[16,17,30–34] Dimensions of polymer-space available for exploration include triblock, comb, or other architectures[32–35] and topologies;[31] alternate chemical moieties such as silicon-containing polymers other than PDMS,[36,37] and oxygen-rich groups such as oligosaccharides and poly(lactic acid);[16] or tailoring polydispersity to modify morphological stability and domain sizes.[38–40] A consequence is that there remains much to be explored synthetically in order to optimize pattern formation, etch selectivity (or resistivity), polymer reactivity, surface energies, Flory-Huggins parameters,[41] LER, and annealing conditions. In particular, there is a persistent analytical barrier that synthetic chemists must overcome in order to readily determine whether their polymeric creations may be applicable to novel DSA applications: they require access to a toolbox capable of analyzing critical features such as the defect density, correlation lengths, and LER of their patterns in order to determine whether their block copolymers have promise. The dearth of accessible tools remains a significant obstacle for the area of directed self-assembly.

Defects themselves also warrant a more in-depth investigation, which can only be achieved by studying defects “in the wild”, in the actual nanopatterns as they progress through various stages of annealing. While simulations can find matches to thin film defect structures,[42–44] automated analyses of defects in block copolymer thin films in an experimental setting allow access to statistical data about the frequency and distribution of various defects. Statistical data is generally inaccessible *via* modeling due to computational limits for defects beyond the simplest examples.[42] Furthermore, for cylindrical block copolymer domains, defects do not always have liquid crystal analogues,[43] rendering past defect-detection methods, originally developed for patterns formed in liquid crystal thin films,[45] inappropriate. Hence identification of structures beyond simple counting of disclinations and dislocations[46] would be advantageous.

Computerized analyses that are widely used to study images of BCP patterns include to determination of periodicity using azimuthally averaged fast Fourier transform (FFT) images,[47] image filtering and regional analysis of domain orientation using FFTs[48] and defect density measurements. Less commonly measured is LER.[49] Rarely, however, are more than one or two methods packaged together in a published work, which leaves unanswered questions since there is typically a trade-off between factors such as defect density, orientation, nanostructure spacing, line-width, line-width roughness (LWR), LER, and correlation length (i.e., grain size). Initially, in the course of investigating the process of microwave annealing of block copolymer thin films, we developed an in-house algorithm to quantify defects in block copolymer thin films, utilizing particle analysis and skeletonization to identify defect features.[28,50,51] Later, while analyzing density doubled cylindrical line patterns, a separate process for measuring the LER was created, which was limited to analyzing nearly-straight segments of nanowire structures.[29]

To remedy the lack of a readily available and straightforward analytical tool, we developed an accessible and free-to-download application for analyzing the defects in BCP thin films using a combined particle and skeleton based analysis of the pattern, called Automated Defect Analysis of Block Copolymers, or ADAblock for short (links to the tool provided in S1 Instructions). The application was constructed using the ImageJ platform, a free, open-source, Java-based image analysis program, which provides a full and easy-to-understand output.[52,53] The tool identifies not only the type(s) of defects found in a sample, but also quantifies the density of defects over a range of length scales, accompanied by additional information regarding LER and LWR, as well as an alternate means of accessing the correlation length. In this work, we screened the ADAblock application against a range of nanopatterns prepared *via* block copolymer self-assembly and show the effects of polymer molecular weight on the defect densities of self-assembled BCP films. Additionally, we demonstrate how ADAblock can simultaneously track LER, defects, and correlation lengths. To our knowledge, no previous work analyzing 2D block copolymer line patterns has brought together data on defects, LER, LWR, and correlation lengths into one application or analysis. We believe that this omission is likely due, in part, to the lack of readily available, widely applicable, easy-to-use tools for analysis, and on occasion, may be a result of selection of the ‘makes-it-look-best metric’, rather than a complete description of pattern quality over larger areas of the sample. In this paper, we show how such data can ideally be combined to better describe line patterns derived from BCP assembly in thin films. Images can be deceiving, and we hope that ADAblock will assist researchers in avoiding pitfalls resulting from performing incomplete defect density analyses.

## Results and Discussion

**Fig 1:**
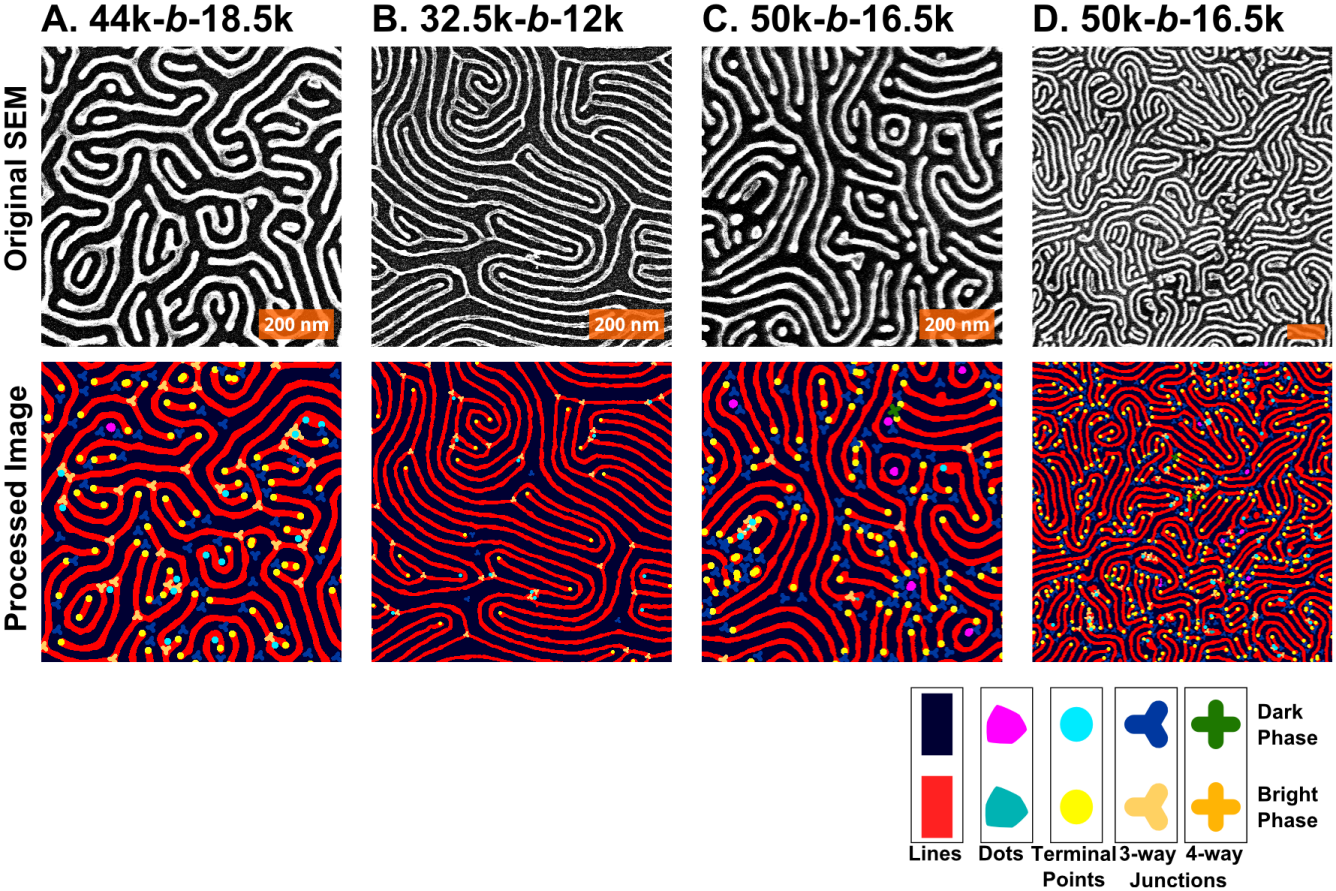
Sample SEM images for Pt line patterns derived from 3 different PS-*b*-P2VP polymers. The following molecular weights correspond to the polystyrene-*block*-poly(2-vinylpyridine) block copolymers used: (A) 44k-*b*-18.5k, (B) 32.5k-*b*-12k, (C & D) 50k*-b*-16.5k. Units are in kg/mol, hence 44k is 44 kg/mol. The first three images are taken at 50,000x magnification; the fourth at 25,000x. The orange scale bars all represent 200 nm.

Examples of four different nanopatterns derived from BCP self-assembled templates are shown in Fig 1. The patterns have been converted into easily visible platinum lines through a well-described platinization of three different PS-*b*-P2VP BCPs;[14,28,29] the Pt nanolines are derived from the P2VP blocks. Although these scanning electron micrographs (SEMs) are similar in appearance, each is subtly different, and thus the question to be posed is how to distinguish one pattern from another and to determine which is more defective. As shown in Fig 1 and Table 1, the pattern in 1A has 30% more defect pairs than the patterns in Fig 1C or 1D. Moreover, the correlation length of 1B is shorter than any of the others (in part due to the shorter period). Additionally, in terms of the line edge roughness (LER), they all appear at first glance to be quite smooth, but the measured roughness of these lines, as summarized in Table 1, would put them out of contention for ITRS targets. With respect to LER, the values of ~ 4 nm are significantly larger than the maximum 0.6 nm suggested,[22] but the feature size here is also ~2x larger than the 10 nm features sought by the ITRS; similarly, the defect density is ~10,000 times higher than ITRS goals. However the present samples lack any features to guide alignment, as is the case with graphoepitaxy, which assists in significantly lowering the observed defect density.[28,44,54]

**Table 1:** Data for each of the four panels in Fig 1, including period, LER, LWR, correlation length, and defect density.

Image	Magnification	Polymer	Period	LER (3σ)	LWR (3σ)	Correlation Length	Defect Density
A	50k	44k-*b*-18.5k	41.4	4.2	6.8	41.5	140
B	50k	32.5k-*b*-12k	29.9	3.3	5.1	93.7	76
C	50k	50k-*b*-16.5k	38.5	5.3	9.2	70.1	187
D	25k	50k-*b*-16.5k	38.6	6.7	10.2	73.8	174

Units are in nm, except for defect density, which is given as defect pairs per μm^2^. Line-edge roughness (LER) is given as three times the standard deviation (3σ) in the edge position, relative to the center of the line; line-width roughness (LWR) is three times the standard deviation (3σ) in the width of the line.

### Outline of the analysis

**Fig 2:**
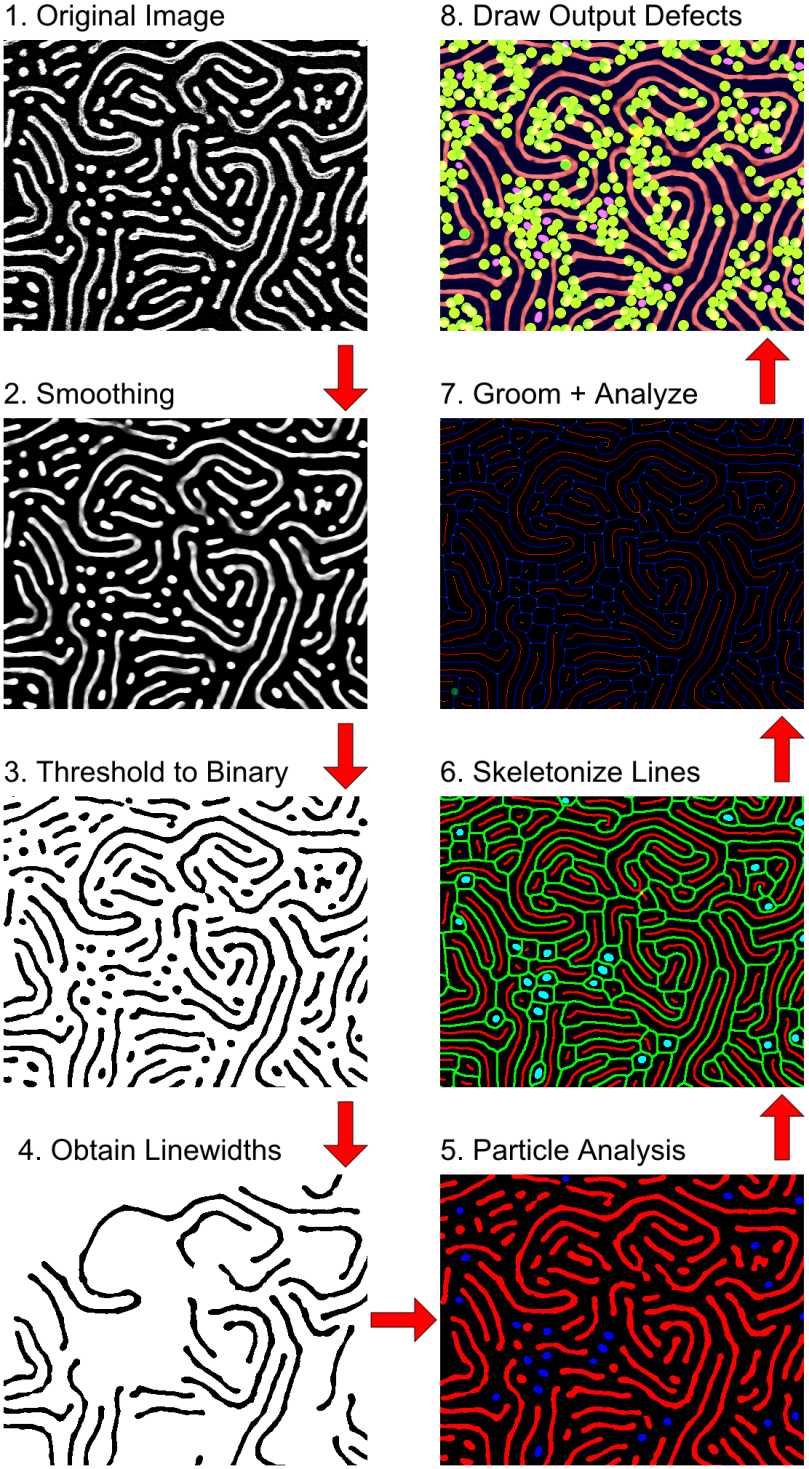
A brief visual outline of the analysis undertaken by the ADAblock application, broken into 8 major stages in sequential order. (1) The original SEM image; (2) Smoothing of the image to reduce noise; (3) Thresholding the image to produce a binary image suitable for particle analysis; (4) Analysis of period and line-widths in order to set parameters in subsequent analyses; (5) Particle analysis of the binary image to find lines and dots; (6) Skeletonization of the lines; (7) Grooming and analysis of the skeletons; and (8) Compiling visual and other data files for output.

The analysis is briefly outlined in Fig 2, breaking down the ADAblock sequence into eight broad stages. Details of each stage are provided in detail, *vide infra*. The first stage is simply the representation of the original SEM image, which must be smoothed to reduce noise, while retaining all features of interest. Next, the smoothed image is thresholded, in order to produce a binary image from which data like area, perimeter, and shape can be determined. Next, the period and line-widths are calculated, followed by particle analysis to determine the shapes of the binary objects and to classify line and dot features. The line features identified are then isolated and converted into a skeleton, from which the connectivity can be determined. This resulting structure is groomed and then analyzed for defects. Lastly, the data is recorded and confirmation images are produced for user inspection. All stages noted in the text correspond to the stages represented diagrammatically in Fig 2.

#### Stages 1 and 2: Input of original image and smoothing

In order to extract information regarding defect density and LER (above, Table 1), a number of factors must be taken into account. Starting with the image itself, basic parameters must be adapted to (1) the image resolution [for instance, determination of how many nanometres are represented by each pixel (nm/px)]; (2) the contrast of the image, which can vary considerably image-to-image and instrument-to-instrument; (3) image noise, which creates artifacts not inherent to the actual structure under investigation; and (4) the period and line-width of the block copolymer. Given the nature of block copolymer patterns typically observed, certain presumptions about the structures observed within the images can be made. To begin, predominant structures within a given image are primarily limited to dots, lines, and meshes. Classification into the basic families of structures in turn constrains certain shape characteristics for the features. Additionally, the period can be defined for a relatively narrow range (e.g. 20 to 40 nm for the samples described here, but modifiable for a given system), as block copolymer samples for a given image data set can be manually selected to those having similar period values. As preliminary background data, the period of a pattern can be obtained *via* azimuthal averaging of the image’s fast Fourier transform before application of ADAblock.[47] The first item, the image resolution, is frequently embedded within the image’s metadata and hence can be called by the program or input by the operator. The preponderance of our BCP pattern images were obtained using a Hitachi S4800 scanning electron microscope (SEM), which provided information in a legend at the bottom of the image; the consistency of this feature also provided a means for automated extraction of resolution parameters.

Combined with its high resolution and high throughput, SEM can be the ideal imaging tool for BCPs, although it does have some drawbacks: For our work, smoothing was necessary due to random noise, charging effects, and edge effects. In the case of SEM images, edges can possess enhanced brightness,[14,28,29,50] and white noise results in speckling of the image with bright and dark pixels. Without some smoothing, such salt-and-pepper noise can result in unwanted extra features. SEM owes much of its brilliance to edge effects, which result in objects protruding from the surface (such as Pt nanowires on Si) appearing much brighter than surrounding substrate.[14,28,29,50] Typically, smoothing images involves trial-and-error, but linking the smoothing to the period of the pattern and the image resolution gives consistent results: Gaussian and/or median filtering are automatically applied with filter radii calculated in proportion to the period of the pattern to avoid under- and over-smoothing. Median filtering typically is best, as it can preserve and even enhance the structure of the line pattern, as shown in S1 Fig

#### Stage 3: Threshold to binary

In order to analyze the pattern, the two phases, each corresponding to one of the blocks, must be clearly identified and separated by thresholding. This assigns each feature in the image to one of the two phases, referred to herein as positive (i.e. bright) and negative (dark). Contrast enhancement and thresholding typically requires manual intervention as well. For SEM and atomic force microscopy (AFM) images, which are typically used for block copolymer thin films and patterns, as well as helium-ion microscopy images, a bimodal histogram is either typical or attainable given the nature of the pattern. Such a bimodal histogram can occur either globally (i.e., over the whole image) or locally (over smaller sub-regions); a suitable thresholding filter can be applied on either scale. Several “auto-local” thresholding plugins are available for ImageJ; analysis of our images typically works best utilizing an auto-local threshold which applies Otsu’s clustering method[55] locally across the image, however, other thresholds implemented in ImageJ are available options.[56] When the surface is not uniformly covered by features (e.g. featureless regions), however, automated thresholding can result in additional artifacts, hence subsequent steps are taken to remove noise and incorrectly phased features.

#### Stage 4: Initial Line-Width Analysis

**Fig 3:**
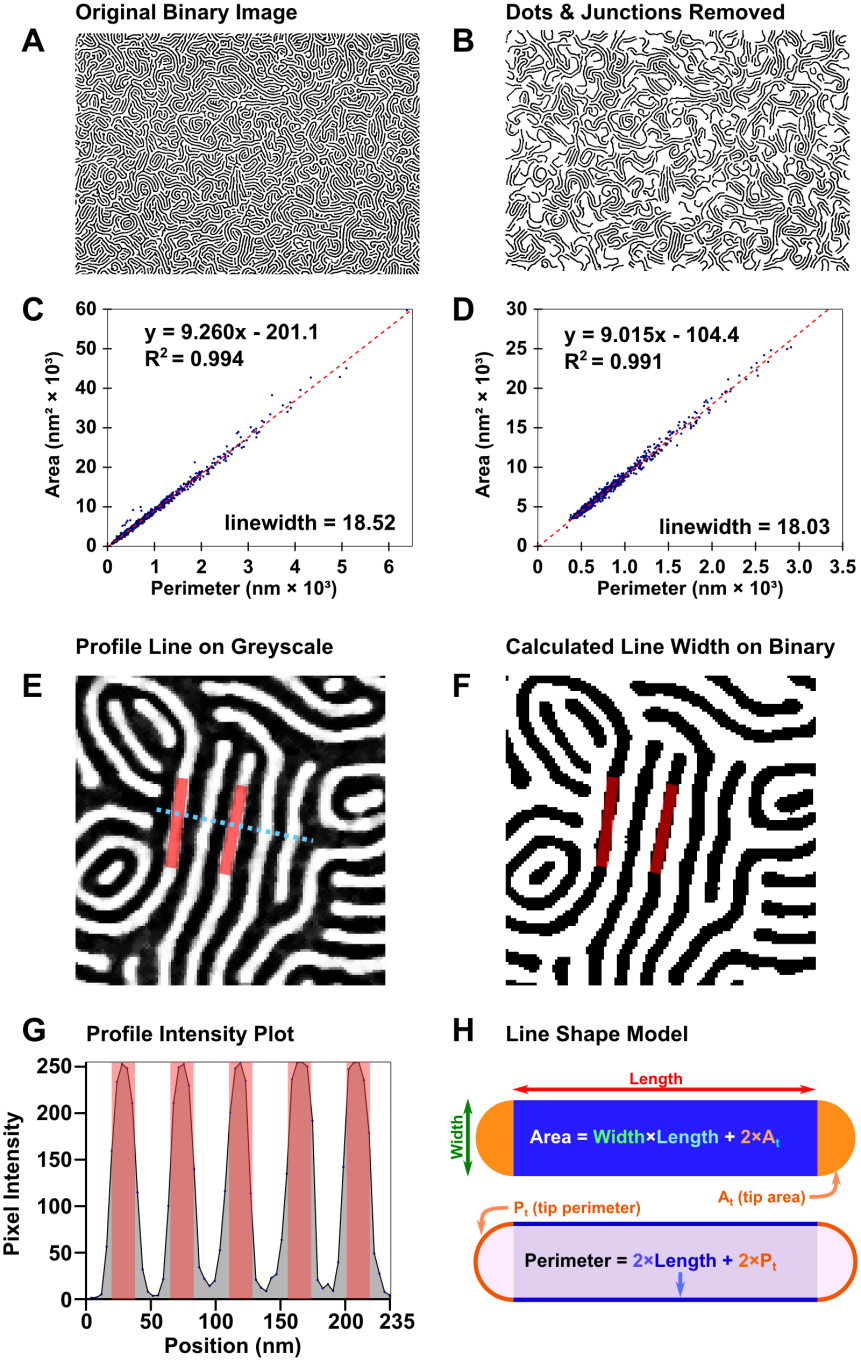
Process for determining line width and period directly from binary patterns. (A) Unmodified binary image of platinized PS(50k)-*b*-P2VP(16.5k) and (B) simplified binary image; (C) fit of particle area as a function of perimeter for the unmodified image and (D) fit for the simplified image. (E) Demonstration that a fit of 18 nm for line-width is reasonable for the filtered greyscale image, (F) the thresholded binary image, and (G) a profile of the filtered image. (H) Line diagram showing the relationship between particle area, perimeter, and length.

The first pieces of data that must be determined are the dimensions. While period can be readily and automatically measured from azimuthally averaged FFT patterns,[47] line-widths and spacings cannot be derived directly from the image of the BCP nanopattern. Profile plots can make for easy manual measurement of these features when patterns are regular and aligned, but that is not always the case. Knowledge of the dimensions is useful, even necessary, in contexts where the pattern is poorly ordered. Particle analysis can measure the area and perimeter of each particle accurately. While Feret measurements (See S2 Fig) work for simple particles, the tortuous nature of BCP “lines” calls for more nuanced measurement. Imagine a spaghetti noodle shape confined in 2D; there exists a relationship between the perimeter of the noodle’s edge and the area covered by the noodle. Using these easily measurable geometric quantities—particle area and perimeter—the width of lines can be calculated, straightness and degree of branching notwithstanding. Provided there are enough lines available, the particle area plotted as a function of perimeter is linear as shown in Fig 3; the slope of the plot is half of the width of the line.

For lines without junctions and only uniform tips, perimeter, *P*, can be broken into

$$P=2P_{t}+2L$$ (1)

where *P*_*t*_ is the perimeter of each tip region (see Fig 3H) and *L* is the length of the main portion of the line. The area can be calculated similarly

$$A=2A_{t}+wL$$ (2)

where *w* is the width and *A*_*t*_ is the area of the line’s tip region. Area as a function of perimeter can be calculated by substituting

$$L=\frac{\left(P-2P_{t}\right)}{2}$$ (3)

into the area equation, giving

$$A\left(P\right)=2A_{t}+\frac{w}{2}\left(P-2P_{t}\right)$$ (4)

$$A\left(P\right)=\frac{w}{2}P+\left(2A_{t}-wP_{t}\right)$$ (5)

As previously stated, the slope is *0*.*5w*. For patterns where junctions rather than terminal points are predominant, *P*_*t*_ becomes zero, and the intercept is positive (due to an additional area term derived from the junction). In practice the contribution of the ends (thus intercepts) is negligible relative to the segment lengths (*L >> w*) for images with junctions. With exclusively semicircular terminal points or triangular junctions, one would expect intercepts of *-0*.*785w* ^*2*^ and *0*.*289w* ^*2*^ respectively. (See SI for calculation.)

Intermediate combinations can be avoided by temporarily excluding junctions and breaking down the binary pattern into smaller, junctionless particles, as shown in Fig 3B. By excluding the junctions, along with excessively small particles and sections of particles on the edge, a better fit can be obtained, providing a better estimate of the line-width. (See S1 Appendix for a schematic depiction of how junction exclusion achieves this.) The value of the intercept in Fig 3A is given as approximately *-200 nm* ^*2*^, which is reasonably close to the predicted value of -250 nm^2^, detailed in S1 Appendix.

Repeating the process for the negative phase, separately, gives a measure of the spacing between lines. Summing the two measurements to approximate the period has, in most cases, been found to come within 5% of the period measured by FFT, usually slightly greater. It appears that this discrepancy may be due, in part, to the particle perimeters being larger than non-discrete analogues, and also due to the approximations made herein. Alternatively, knowledge of the line-width and the period would give the line spacing by difference.

#### Stage 5: Particle analysis

In the course of annealing spin-coated BCP thin films, the pattern may evolve from a dot pattern, or similarly disconnected collection of features, into an array of lines, with numerous defect-rich intermediate states. The binary image can be analyzed using ImageJ’s built-in particle analysis routine to determine characteristics of particle size and shape descriptors such as circularity and Feret measurements, mean pixel values, and relationships to the image boundary for each feature. Particle analysis is done separately for the positive and negative phases in order to access all the features. Particle analysis data is then used to separate dots (or other objects), which cannot be accurately treated as lines, and identifies them as a specific type of defect. It can also provide information on the evolution of particles in the course of the annealing process (e.g. increases in the average size or length of lines). Moreover, the creation of a binary pattern further enables distinction between noise and misclassified particles.

#### Stage 6: Skeletonization

The most effective means to analyze the topology of lines and meshes is *via* analysing the connectivity of the pattern by creating a skeleton of it. Skeletonization reduces lines or meshes to binary objects which maintains the connectivity of the original by “thinning” the pattern to create a simplified, single-pixel-wide version of the shape, suitable for pixel-by-pixel analysis. Skeletonization algorithms and skeleton analysis has been widely used in other fields to study the topology of structures, from text recognition algorithms in computer science to numerous subfields in biology, including bone analysis (“bonej”),[57] and studying the structure of neurons, as well as for the recognition of typographic characters. In all cases, skeletonization is used to simplify collections of interconnected shapes and objects into networks to study their properties. Although other algorithms do exist, the default technique is implemented in ImageJ:[58] the skeletonize function in ImageJ uses a lookup table to progressively thin the structure based on each pixel’s 3x3 neighbourhood, leaving a 1-pixel wide topological skeleton.[58] At least two other groups have applied skeletonization as a means to interpret BCP thin film patterns.[59,60] Rehse and coworkers utilized skeletonization of *one phase* of the polymer pattern to study frame-to-frame correlations between junctions as a measure of BCP dynamics;[59] their work followed that of Scherdel[60] and Vigild[61] who used 3D interpenetrating skeletons to describe gyroidal phases.

**Fig 4:**
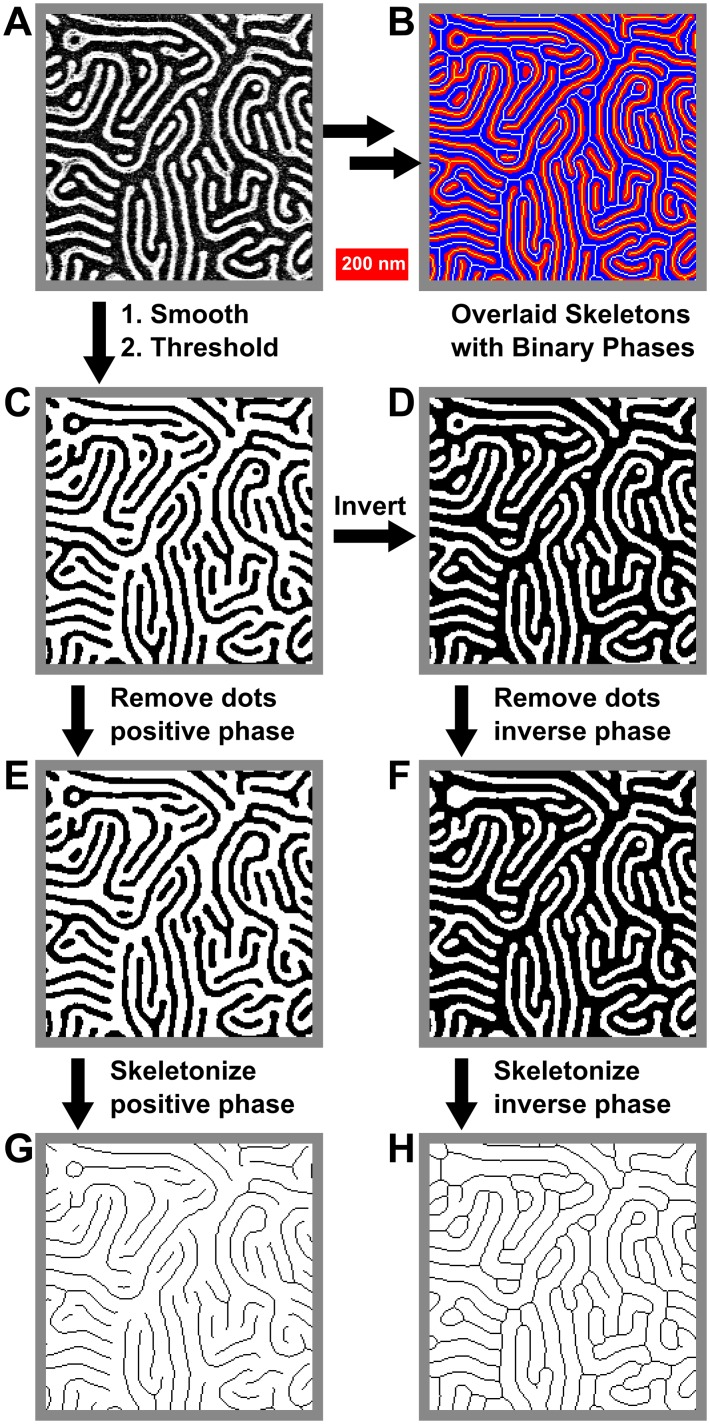
Process for the skeletonization of both positive and negative phases of a binary image. (A) Original image. (B) Overlay of binary and skeletonised images showing retained connectivity. (C) Binary image. (D) Inverted binary image. (E) and (F) Processed images (C) and (D), with dots in respective phases removed. (G) and (H) Skeleton images derived from (E) and (F). Images are all 735 nm × 735 nm.

The skeletonization process itself is quite straightforward, as shown in Fig 4: to skeletonize a binary image (Fig 4C), first dots are removed (Fig 4E), leaving only line features, then the image is thinned as described above (Fig 4G). It is important to skeletonize both phases of the image, so the original binary image is then inverted (Fig 4D), dots from the negative phase are removed (Fig 4F), and the inverted image skeletonized (Fig 4F). Overlaying the skeletons with the binary images (Fig 4B) shows that skeletonization indeed preserves the connectivity found in the original image (Fig 4A).

**Fig 5:**
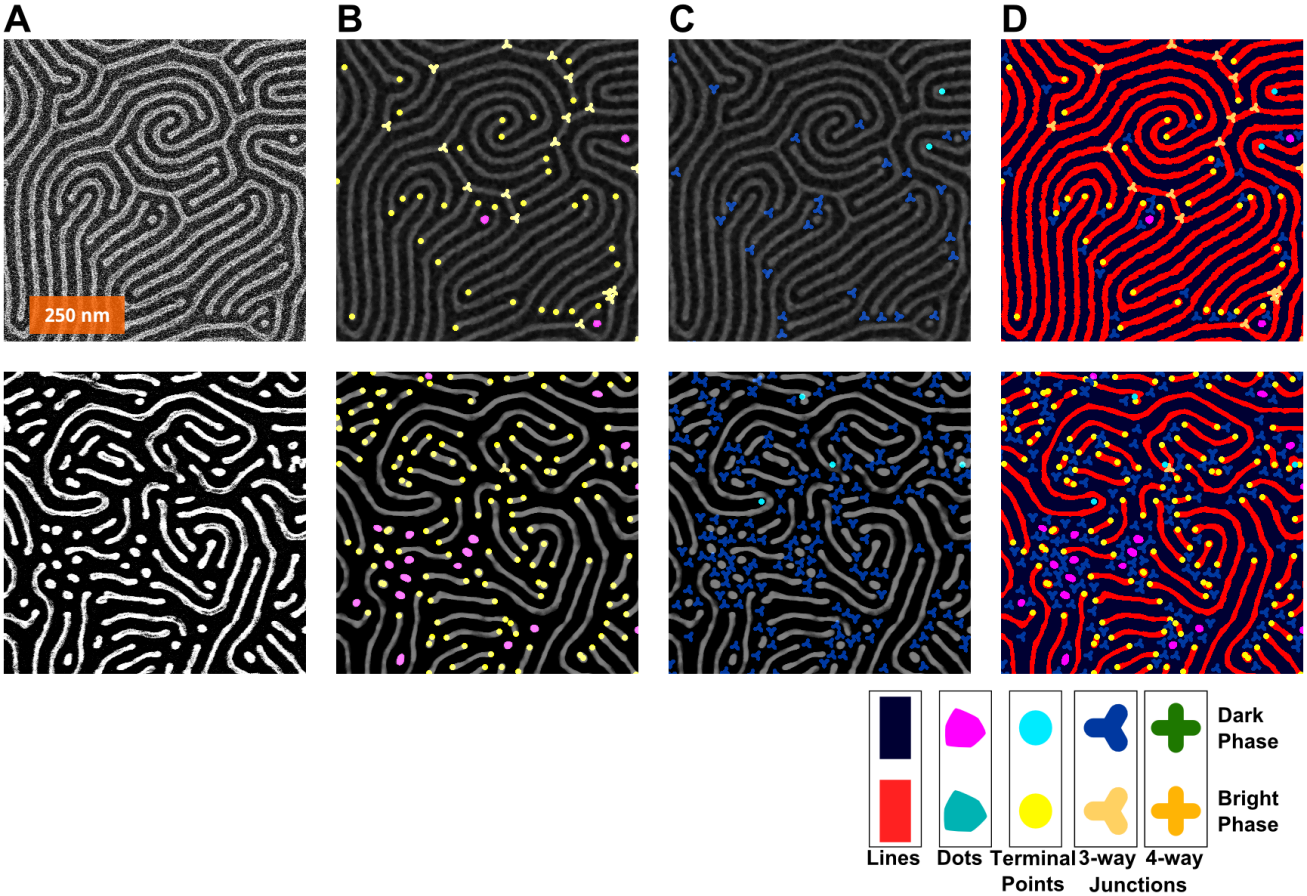
How the defects, depending on phase (bright = “positive”; dark = “negative”), tend to be of different types. Analysis of two images are shown in parallel with corresponding images in two columns. (A) The original images. (B) Defects in the positive phase marked. (C) Defects in the negative phase marked. (D) All defects. Legend at the bottom shows colours and shapes used for each feature: Bright phase: red lines, teal dots, yellow circles at terminal points, and 3- and 4-connected junctions. Dark phase: navy blue lines, magenta dots, aqua terminal points, and 3- and 4-connected junctions represented by shapes with an equal number of branches.

In order to actually access the defects in striped BCP patterns, it is necessary to investigate each phase separately, thus requiring parallel particle analysis and skeletonization of each phase. It is worth noting that for BCPs, certain kinds of defects will prefer one phase to the other, resulting in a surplus of terminal points or junctions in either phase. This tends to limit the frequency of spatially paired defects. Fig 5 shows some examples of this effect. In the images on the left of Fig 5B, there are ample junctions in the positive phase; in the images on the right, there are almost no junctions in the positive phase, despite having more defects overall. Similarly in Fig 5C, there is a greater proportion of terminals in the negative phase than in the corresponding image on the right side. Such features contribute to the topology of the pattern, which can be affected by the means of annealing.[62]

#### Stage 7: Groom and analyze

This stage is the most complicated, and it is divided up into separate sections, (a)-(d) based upon the type of analysis.

**Fig 6:**
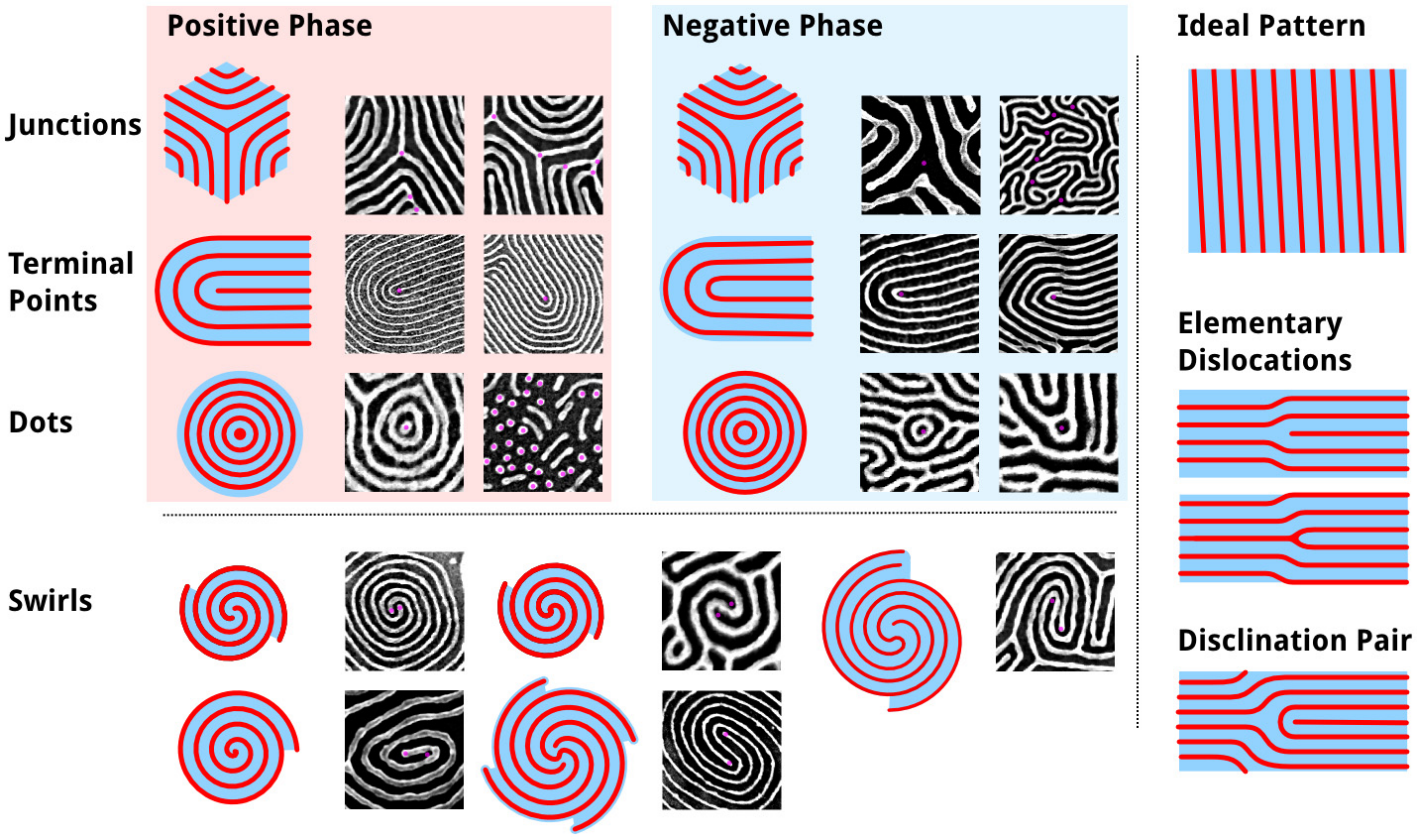
Table of topological defect components typically found in BCP thin film nanopatterns. Shown are each major type of component defect, as exists in either the positive (e.g. P2VP) phase or the negative (e.g. PS) phase. For each, 3-branch junctions, terminal points, and dots, examples are given with defects highlighted by a magenta dot. This analysis is done relative to an ideal striped pattern without any interrupting features, save for the edge of the image.

*(a) Locating defects*. The ideal, defect-free line pattern derived from lamellar or cylindrical domains of block copolymers, consists of perfectly parallel straight lines extending across the entire substrate without interruption, as by breaks or junctions in the lines. It is with respect to this ideal that topological defects are defined. The analogy between block copolymers and liquid crystals (nematic and lyotropic phases in particular) inspired previous defect analyses[63] utilizing winding numbers to identify and measure topological defects. While this does work in principle, and many previous analyses have utilized it and other defect-detection methods,[46,59] these methods typically are published without full working details or code. Kléman suggested in 1983 that defects in two-dimensional line patterns could be simplified to junctions, terminal points, and dots, shown in Fig 6, rather than the more conventional approach using winding numbers to determine the type of defect.[64] While such methods correctly describe the type of defect,[46] high levels of defects and variability of the patterns, including variations in line-width (or LER) can make it difficult to correctly identify and quantify defects. One author noted an order of magnitude change in the density of defects for one particular image depending on the amount of Gaussian filtering applied; the filtering parameters ultimately selected appeared to be arbitrary.[45] Furthermore, images depicting disclinations and dislocations suggest imprecision in the identification of closely associated dislocations.[46] Moreover, while such methods provide the magnitude of each defect, they do not provide information about the connectivity of defects or orientation of surrounding features.

Many frequently encountered defect structures are not isolated dislocations (junction-terminal point pairs immediately adjacent) or disclinations (either terminal points or junctions), but are part of more complex defect structures. These structures can be broken down into component dots, junctions, and terminal points, the elementary components of defects shown in Fig 6. Dots can be determined best using the particle analysis data, so from the skeleton analysis we locate and characterize the junctions and terminal points.

**Fig 7:**
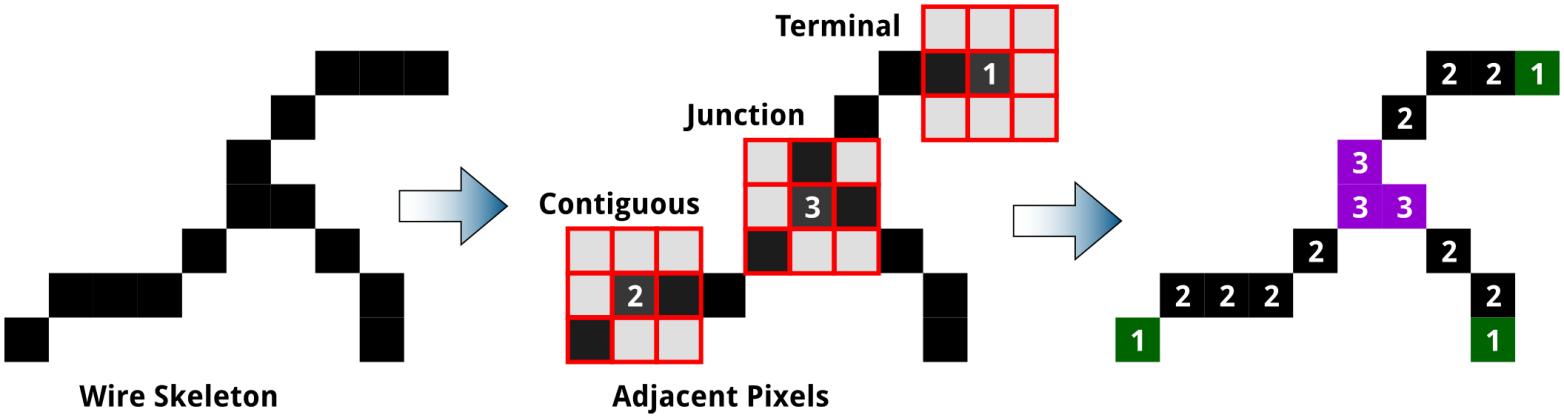
Pixels of a typical junction and three associated terminal points showing the counting of adjacent skeleton pixels. Highlighted are pixels representing (i) terminal points at the end of the line or branch, each adjacent to only 1 pixel, (ii) contiguous points along the line, each with 2 neighbour pixels, and (iii) junction points where three or more branches meet, having 3 or more neighbour pixels. Similar to minesweeper games, the number of adjacent pixels determines the value of each skeleton pixel.

At this point in the analysis, the skeleton is a binary object where lines are represented by a series of 2-connected pixels in any of the 8 directions; terminal points are singly-connected pixels; and junctions occur where more than 2 pixels are neighbouring a given pixel. Dots do not, however, always reduce to single-pixel objects and hence they are treated separately. Furthermore, junctions exist in numerous possible configurations, often with multiple (3+)-connected pixels per junction, hence there will not be a one-to-one correspondence between junction pixels and either the number of junctions or junction types (see Fig 7). Identification of defects is done in a manner, which is, in essence, analogous to playing the minesweeper-type[65] games: by counting the number of skeleton pixels adjacent to any given pixel that is part of the skeleton, thus providing a connectivity value for that pixel, as shown in Fig 7.

At its simplest, any connection or disconnection that breaks the 2-connected topology of the skeleton, resulting in a new local topology (or connectedness), is a defect with a corresponding value. For junction points (JP), with each additional branch beyond two (which, on its own, would constitute a line without topological defect), the defect increases in magnitude by ½:

$$n_{jp}=-½\left(B-2\right)$$ (6)

where B is the number of branches. Typically junctions come with only 3 branches, but 4-way, or even 5-way, intersections can be found, on occasion, between clusters of dots or other complex features. A 4-way intersection would be *n*_*jp*_ *= -1*, which can be imagined as being derived from two adjacent junctions, each with *n*_*jp*_ *= -½*, with a common line-segment, where the intervening line segment’s length decreases to zero; the same approach can be generalized for any number of additional line segments (as shown in S3 Fig).

Terminal points (TP) possess only one configuration, hence their value is assigned:

$$n_{tp}=+½$$ (7)

Dots can be considered as a line with two terminal points, collapsed to a single point (as depicted in S3 Fig), hence their value is twice that of a terminal point:

$$n_{dot}=2n_{tp}=2\left(+½\right)=+1$$ (8)

Other more complex structures, such as spirals (containing terminal point dislocations) can be counted *via* their component structures in this regard. Large, solid spots in the bright phase or large regions without any pattern (i.e., large spots in the dark phase) possibly formed due wetting (or other causes) may exist, these regions may be treated as dots with radiating arms, however, we found it was more effective to separate the core of the dot prior to skeletonization. The result treats the dot as a kind of enlarged junction, with defects existing only at the periphery.

For all defects to be counted, skeletons for both phases must be generated and connectivity analyzed separately. This raises an important point for defect analysis: that defects exist in a particular phase. The phase dependency of defects has not been sufficiently explored, however hints are seen in the literature, as it directly controls the topology of a system.[66] The dual phase analysis brings about an addition rule for description of the system: on average, the sum of all defects in the pattern should be zero. Alternatively, this can be stated that every defect is “paired”, hence for every junction there is a terminal point; Moreover, for every dot, there will be two junctions: consider this an analogue of a unit cell (see S7 Fig), as two defect pairs are produced if spontaneously generated and two are required to cancel out through annihilation.[46] Typically there exists a small imbalance between the two measures, which others have observed as well.[45] The pairing of defects does not imply however that the number of defects in the two phases of the block copolymer will be equal.

Skeletons also provide a description of the connectivity of defects, which merits further exploration. Defects can also be associated with particles using this method, but perhaps the greatest benefit is derived from the ability to search for and positively identify particular clusters of defects. One such example is an H-junction, which results from a break in the line or a bridging of two adjacent lines, shown in S4 Fig These junctions are supposedly not the result of a defect in the actual thin film structure, but result from (a) incomplete metallization or other means of pattern transfer, (b) image noise, or (c) the smoothing-thresholding process. Hence it may be prudent to recognize them and count them separately or to “correct” such errors in the binary image itself.

**Fig 8:**
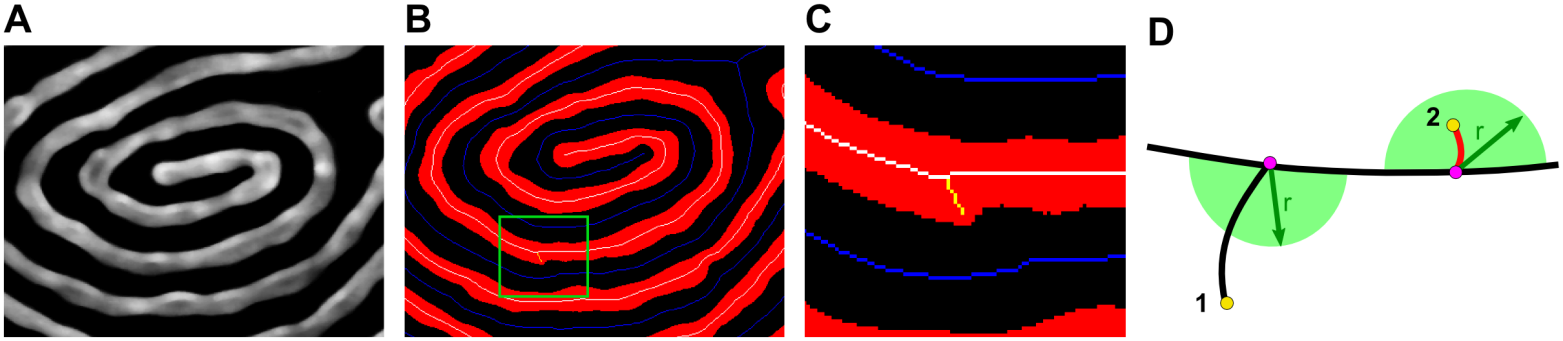
Grooming the skeleton to remove junctions formed as an artifact from variations in line width or from edge effects. (A) Image of metallized PS(50k)-*b*-P2VP(16.5k) nanowire. (B) Image of skeletonized image, with positive lines in red and skeleton in white, and negative lines in black and skeleton in blue. (C) Detail of region identified by green box in (B), showing a branch, yellow, trimmed from the skeleton. (D) Schematic showing radius-based trimming of branches: (1) a branch that exceeds the radius does not undergo trimming and (2) a branch that terminates within the radius is trimmed.

*(b) Grooming the Skeleton*. Grooming the skeleton consists of trimming away short branches, which may result as artifacts from small “bumps” on the edge of a line. With the dimensions determined in Stage 5, we can create a metric to selectively prune away any branches resulting from variations in line-width or simply from sharp points or edge effects that can influence the skeletonization algorithm. It may be a point for philosophical debate what constitutes a branch, justifying a junction and terminal point, but objectivity can be introduced by basing the grooming procedure on the measured LER. For this purpose, any end point separated from a junction by less than 1.5 × line-width (for a given phase) is considered roughness, rather than an additional defect pair, and is hence pruned, as shown by the example in Fig 8.

Because any image represents a finite sample of a larger structure, defects at image edges must be carefully treated. Depending on the resolution of the image and the domain size of the block copolymer, these can for smaller images, represent a significant fraction of defects; additionally, in otherwise low-defect patterns, features cut off at the edge may appear as additional defects. In particular, three rules must be applied:

1. Any “dot” (or sufficiently small object without junctions) touching 2 edges is not a defect. (See S4 Fig)
2. Lines that run roughly parallel to the edge, touching at all times, are not defects.
3. Lines that terminate at the edge of an image are not defects, as it is not a true terminal point.

The third rule requires some manipulation of skeleton points & component terminal points near edges, as ImageJ’s native skeletonize algorithm can produce limited edge artifacts. What these rules do not address is particulate matter; other analysis methods tend to default to manual identification or require equipment unavailable to most researchers.[54]

**Fig 9:**
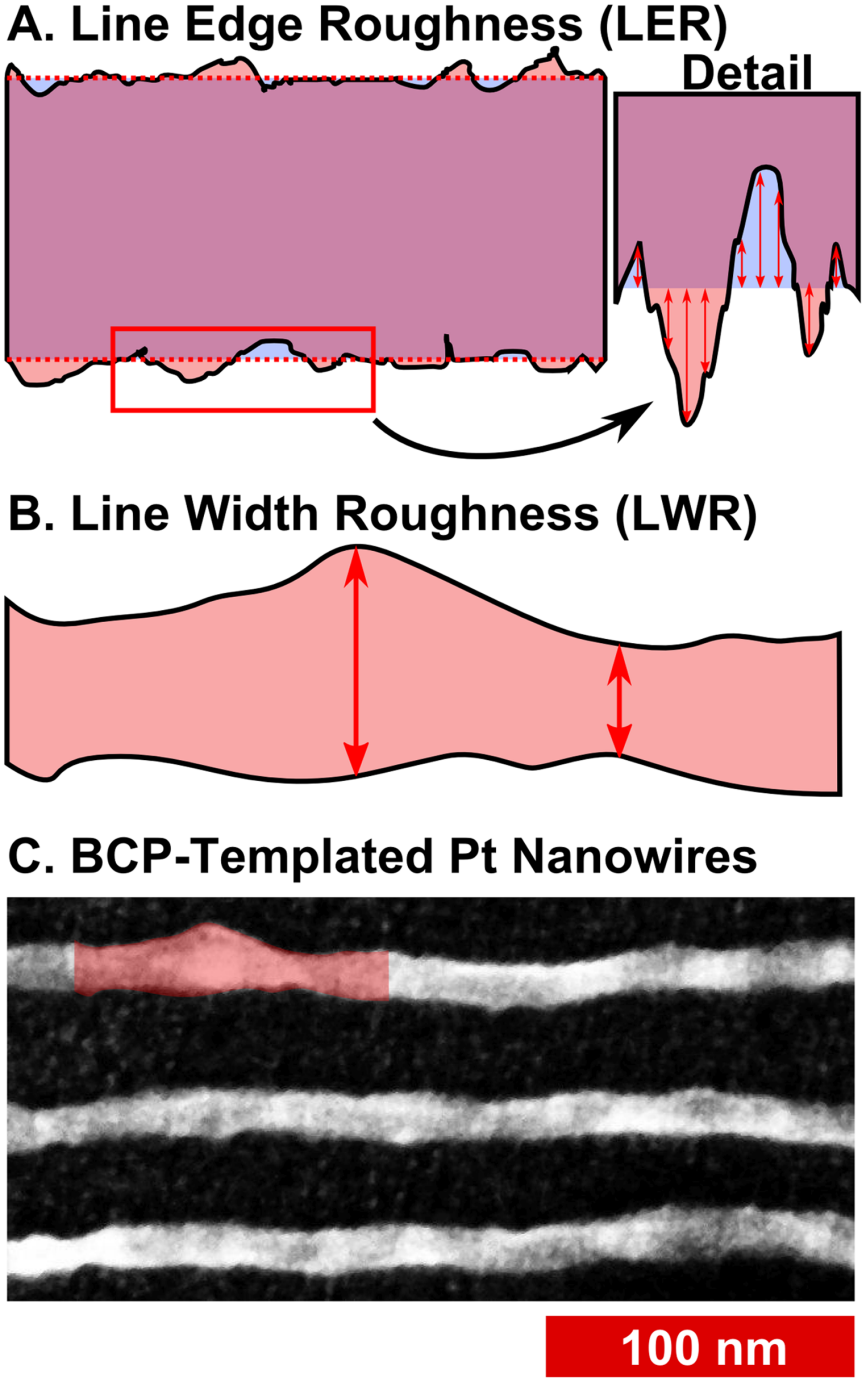
Diagrams depicting measurement of line-edge roughness and line-width roughness. (A) Sketch to conceptually demonstrate line edge roughness, where the variation in edge position of the line (shown in rose with black edge) varies with respect to the ideal (shown overlaid in blue) or, in this case, the average edge position. Each individual displacement is measured with respect to the average, and the LER calculated as 3 times the standard deviation. (B) Sketch of line-width roughness, which is the variation in line-width. The sketch is adapted from the bulges and pinches shown in the SEM image below. (C) SEM image of block copolymer templated Pt nanowires on a Si wafer, using PS(44k)-*b*-P2VP(18.5k), annealed at 200°C for 20 minutes.

*(c) Line-Edge Roughness and Line-Width Roughness*. One of the chief questions posed for BCP lithography is whether lines can be produced with sufficient uniformity and with smooth edges. LER measures the variation in the position of the edge of a line, which can have different frequency components, leading to undulation of the edge and variation in the width of the line, or LWR (Fig 9). The variation in position is measured as the standard deviation in the position of the edge, and LER is reported as 3σ. Such variations are deleterious for circuit elements: For transistor gate features with widths < 85 nm, line roughness causes significant variations in the off-current, as well as affecting threshold voltages.[67,68] For nanometre-scale interconnects, line roughness increases both resistance and capacitance,[69,70] resulting in degraded transistor performance.

**Fig 10:**
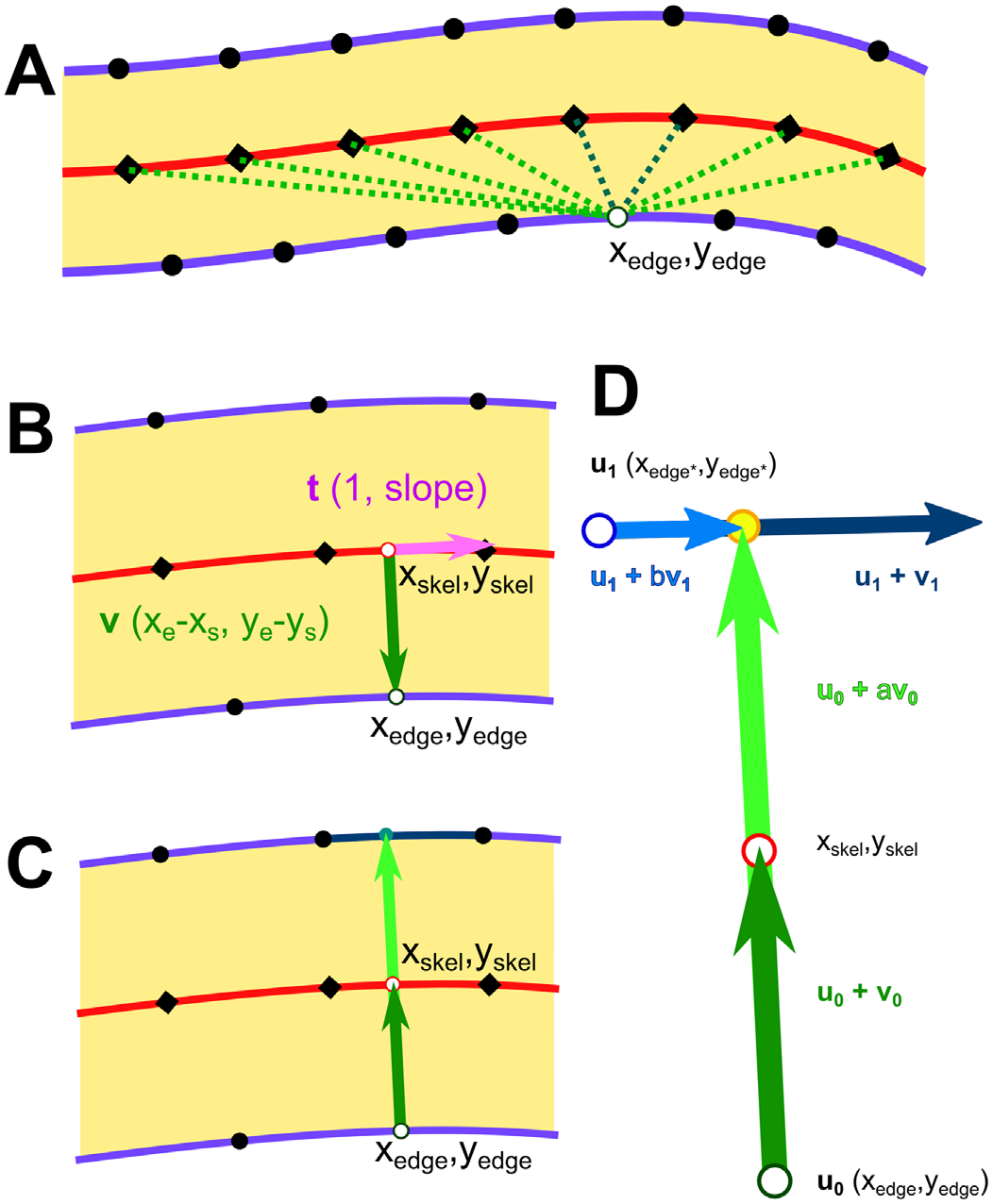
Diagram showing relationship between line edge points, skeleton points, and the vectors used to determine edge positions and line-widths for LER and LWR. (A) Outline of a line, showing edge points (black dots) and skeleton points (black diamonds) on the centre line. One edge point (x_edge_,y_edge_) is selected and distances to nearest skeleton points are checked. (B) Interpolation to nearest orthogonal point from the edge point to a point on the skeleton segment. (C) Extension of edge-to-skeleton vector to intersection with transverse edge segment. (D) Expanded, with parameterization as scalable, intersecting vectors.

The line roughness of block copolymer nanostructures has been considered theoretically and has been shown to depend on χN,[71–73] and polymer polydispersity;[73] results have suggested that the Flory-Huggins χ parameter may need to be increased by a factor of 3 to 4, relative to that of PS-*b*-PMMA,[72] in order to decrease LER sufficiently to accommodate ITRS targets.[74] It has been specifically noted that there are few reports on the topic of LER/LWR in the literature;[49] typically, the actual position of the edge is measured relative to the ideal or average edge position for straight or aligned lithographic patterns. In order to achieve the same measurements for block copolymers, films aligned *via* graphoepitaxy would typically be required in order to have linear lines representing ideal edges. However, we[29,75] and others[76] have taken the approach of measuring LER for unaligned patterns. One may measure edge positions relative to the centre of the line, rather than with respect to a linear ideal edge position; the standard deviation in the edge position will be the same either way. As lines get narrower, however, the influence of pixel position can begin to slightly increase the measured LER, up to 0.5 nm in our previous work using high resolution (ca. 100,000x) BCP patterns. We mitigate this, in part, by smoothing both the centre line of the skeleton and the outer edge, while constraining the positions of the edge points. Edge-to-skeleton distances are determined for all points on the smoothed line edge, matching with the nearest points (shown in Fig 10A) on the smoothed skeleton line which satisfy:

$$\left(x_{edge}-x_{skel}\right)+slope_{skel}\left(y_{edge}-y_{skel}\right)=0$$ (9)

As derived from the dot product of the vector on the edge-to-skeleton distance and the orthogonal vector *(1*, *slope)* of the skeleton at that point, an interpolated point on the skeleton can be obtained (shown in Fig 10B).

Line-width measurements can be made in conjunction with edge-to-skeleton measurements by finding a line segment on the opposing edge, which is intersected by the vector made between the edge point and skeleton point of the previous step (shown in Fig 10C). The solution exists at a point on the line segment formed by the vector between the edge *(x*_*edge*_, *y*_*edge*_ *)* and the skeleton *(x*_*skel*_, *y*_*skel*_ *)* is scaled by a factor, *a*, and on the line segment formed by the vector between two consecutive points on the transverse edge *(x*_*trans1*_, *y*_*trans1*_ *) & (x*_*trans2*_, *y*_*trans2*_ *)*, scaled by a factor, *b* (shown in Fig 10D). Provided that the two vectors are not parallel, the equations[77] for the scalars, *a* and *b*, are:

$$d=\;(x_{trans2}-x_{trans1})\left(y_{skel}-y_{edge}\right)-(x_{skel}-x_{edge})\left(y_{trans2}-y_{trans1}\right)$$ (10)

$$a=d^{-1}\left(\left(x_{edge}-x_{trans1}\right)\left(y_{trans2}-y_{trans1}\right)-\left(y_{edge}-y_{trans1}\right)\left(x_{trans2}-x_{trans1}\right)\right)$$ (11)

$$b=d^{-1}\left(\left(x_{edge}-x_{trans1}\right)\left(y_{skel}-y_{edge}\right)-\left(y_{edge}-y_{trans1}\right)\left(x_{skel}-x_{edge}\right)\right)$$ (12)

**Fig 11:**
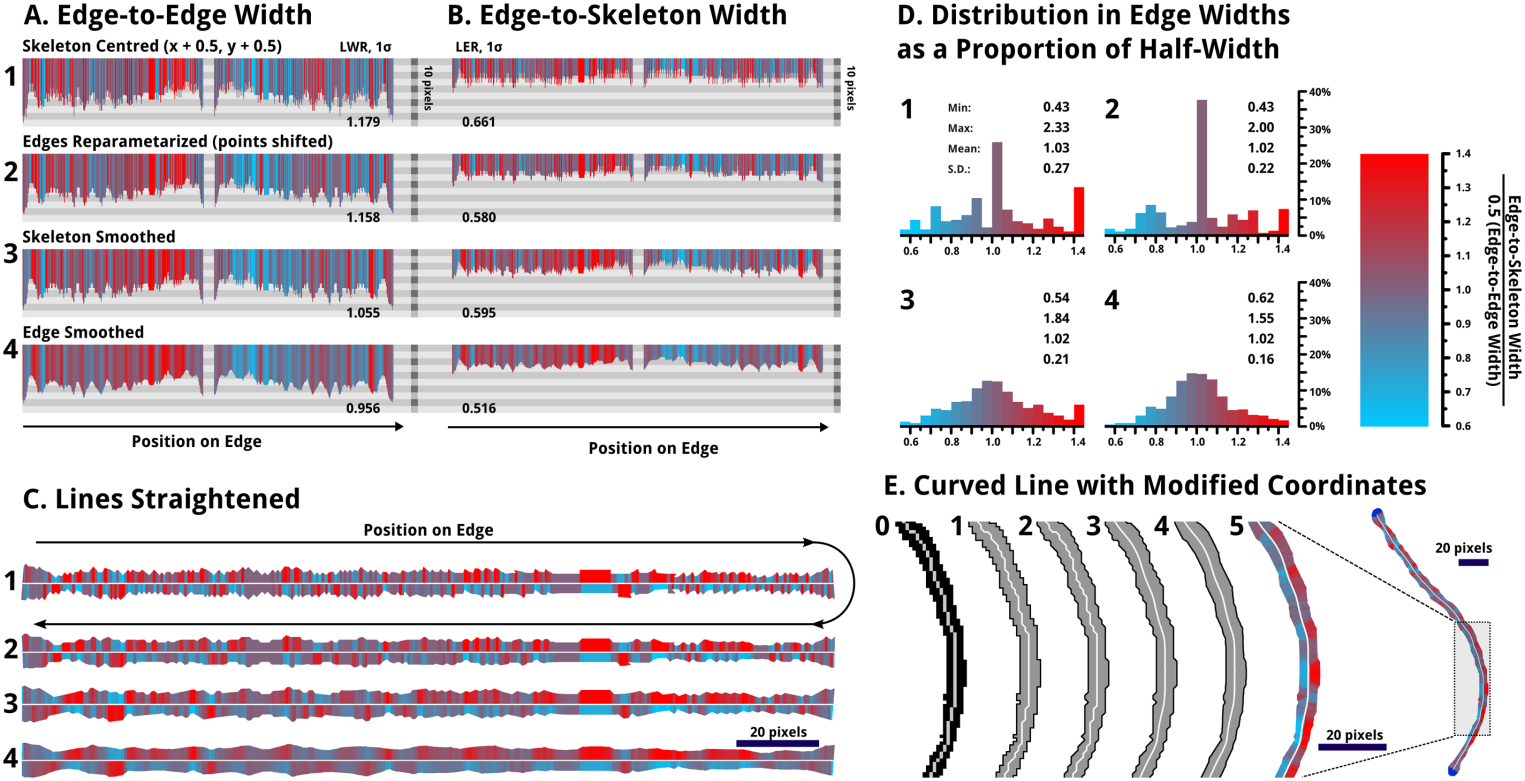
The smoothing process used to partially eliminate roughness resulting from pixelation of the lines. The labels 1, 2, 3, and 4 mark the line subject to each of the four stages of smoothing described. All images with the cyan-to-red colour scheme show the relative width of the opposite side of the line, from the skeleton centre, to the edge; if a side is wider in proportion it is shown in red; narrower is shown in cyan. A colour scale is given provided. (A) The top left shows the edge-to-edge width, following both sides of the edge of the line (C1), hence it is roughly symmetric; (B) the edge-to-skeleton widths are plotted similarly, but with roughly half of the displacement. (C) Next, the lines are shown replotted in a straightened fashion. Note that the lengths have been scaled to be equal, as smoothing of the skeleton shortens the length measured along the skeleton, as expected, due to smaller point-to-point displacements. In the above 3 cases, the more smoothed lines show smaller variations in colour. (D) The histograms represent the edge to skeleton widths relative to the half widths for each point. € Last, the original skeleton (0), along with the 4 stages of smoothing (1,2,3,4) are shown for a re-drawn line, along with a the distribution of edge widths *via* colouration (5).

An intersection is considered valid when *1 < a < 4*, indicating that the side opposite would have a width ranging from 0 to 3 times the width of the first side. The limit, *a < 4*, prevents identification of points on parallel segments, as with a hairpin, from being identified as valid; typically the period is on the order of 2 times the width of a given line, hence 4 times the half-width of a line. In practice, the values of *a* are in the range *1*.*5 < a < 2*.*5*, as can be seen typified in Fig 11 *via* the histograms. The second limit for valid points is that *0 ≤ b ≥ 1*, which ensures that the point of intersection is within the line segment formed by the two consecutive edge points.

In order to obtain reasonable measurements of LER and LWR, the blocky structures of binary lines and skeletons need to be smoothed. The smoothing process, which we have utilized here, involves 4 stages:

- Centring of the skeleton points by adding 0.5 px to each x and y coordinate. This accounts for the slight truncation from the skeletonization process and makes the edge-to-skeleton distances more equidistant on each side.
- Shifting all edge points to the midpoints between consecutive points. This averaging reduces roughness introduced by the shape of individual pixels.
- Smoothing the skeleton by iteratively averaging the positions of points, while limiting the displacement to within 0.25 pixels. This provides a smooth, continuous, reasonably centred skeleton line.
- Smoothing the edges likewise provides a smooth edge while maintaining the shape and deviations in width, from which roughness can be measured.

Fig 11 shows the data for a single line as it is modified by each of these four smoothing processes (A, B, C, D). By the fourth stage (D), the data shows considerably less noise. In particular, the histograms of edge widths, depicting which edge is further from the skeleton, for each point on the edge, begins to approach a normal distribution, as one would expect for a line with random variations in width. Visually, the line becomes sufficiently smooth that pixels are no longer apparent, while variations in width are in keeping with the original image, and the sequential widths and edge positions measured do not have large point-to-point changes in displacement. While the skeletonization algorithm is largely effective in finding the centre line, it is imperfect. In particular for lines with pixelated widths less than 7 pixels, the centre will tend to be skewed preferentially depending on the orientation of the line. However this does not affect LWR measurements and smoothing does help to limit the impact on LER.

In order for BCPs to be relevant in industrial manufacturing, they must achieve a low frequency LWR (3σ) of 1.1 nm on features 16 nm wide; in order to “significantly exceed” conventional lithography, the patterns would need to be better than 0.6 nm LWR on features 9 nm wide.[74] Presently our best measured samples have a LER (3σ) of ~ 2 to 3 nm,[29] however, no aspect of the process has, as of yet, been explored with respect to minimizing LER or LWR. To avoid the local effects of junctions and to increase the speed of the calculation, the lines are modified, as shown in Fig 3B, to render all lines junction-free. Additionally, points where lines contact image edges are selectively modified, erasing large contacts, to prevent any effects of the image edge.

*(d) Correlation Lengths & Order Parameters*. Correlation lengths (or orientational persistence lengths) are typically calculated for large images, often with low resolution (pixels/nm), by subdividing the area into overlapping squares, for which azimuthal angles are derived from two-dimensional FFTs of each region.[78] Lack of clarity for such images sometimes necessitates filtering in order to avoid disordered regions. In this work we implemented an alternative means of determining the 2D correlation function using the skeletonized lines. Skeletons are groomed to remove junctions and loops are broken to provide isolated lines. Orientation along the skeletonized lines can be calculated using a rolling average of each line’s tangent to provide smoothly varying angles along the lines. In a typical image, there can be over 20000 points in the lines; calculating the correlation length using every point is feasible, however for expediency, the set of points can be downsampled or randomly sampled to a smaller set of ~ 4000 points, which provides faster calculation with minimal trade-off in terms of accuracy. From the set of orientation angles, φ(**r**), the correlation function, C(**r**-**r**’), can be calculated.

$$C\left(r-r^{\prime }\right)=\langle cos\left[2\left\{\varphi \left(r\right)-\varphi \left(r'\right)\right\}\right]\rangle$$ (13)

Advantages of this method include ease of applicability to higher resolution, smaller-area images and images with disordered regions where, due to defects, line segments are particularly short, and φ(**r**) might not be determinable *via* FFT. This is demonstrated in Fig 11.

The correlation function is fit using an exponential function,

$$C\left(r-r^{\prime }\right)=exp^{\frac{-r}{\kappa }}$$ (14)

where κ is the *correlation length*, a characteristic measure of the degree of ordering in the film, which describes the average distance over which orientational order is preserved. The correlation length should be proportionate to the grain size, as illustrated by the circles in Fig 11, which are approximately keeping in proportion with the domains visible in the orientationally-colour-mapped pattern image. However the circles are unquestionably smaller than the observed domains.

**Fig 12:**
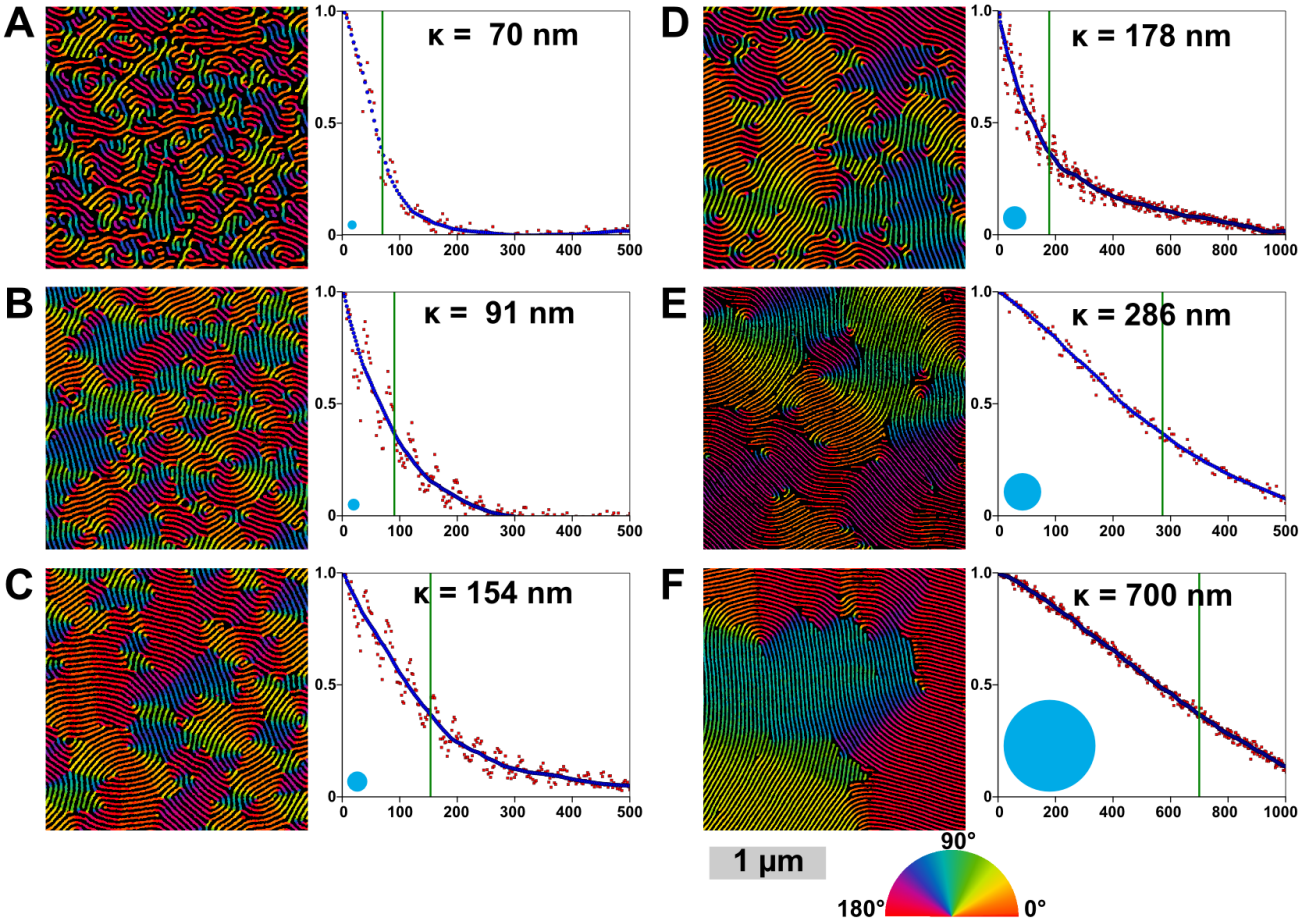
Correlation lengths and orientation maps for six SEM images of metallized PS-*b*-P2VP (50k-*b*-16.5k, 44k-*b*-18.5k, and 32.5k-*b*-12k) patterns with different degrees of thermal annealing. SEMs are shown in false colour to display the angle of each wire as used in the calculation of the correlation functions, shown right. The raw correlation data is shown in red, the smoothed data is blue, and the calculated correlation length (κ) is marked with a green line and noted on each plot. Beside each image is a blue circle whose radius is equal to the correlation length, as the correlation length is often given as a measure of average grain size. Each image is shown cropped here to ~2 μm wide. The scale bar is 1 μm. (See S5 Fig for full images). The labels (A-F) correspond to the same labelled images in Fig 14.

One disadvantage of this method of determining κ *via* skeletonization is that one observes a periodic variation (corresponding to the periodicity of the pattern) in the correlation function, as shown in Fig 12. This periodic variation is a result of features separated by non-integer line spacings tending toward greater disorder than points separated by integer spacings. This appears to be due to influence by neighbouring defects. The large undulation in the curve can be partially compensated by using both the positive phase and negative phase skeletons (thus reducing the period and amplitude of the variation, however exclusion of non-line areas may be necessary), by binning measurements, as is typically done in FFT-based methods,[79,80] or by smoothing, as we apply in the algorithm.

Herman’s orientational parameter, S,[81] gives a measure of how uniformly oriented the lines within an image frame are. It can also be readily calculated using the set of orientational data:

$$S_{2D}\left\{0,1\right\}=2{\left[cos\left(\varphi \right)\right]}^{2}-1$$ (15)

The reference angle can be set as the average orientation for the whole image, thus giving the best orientation parameter for a disordered image. Because it is widely used, we implemented this calculation into our code, however, Herman’s orientation parameter tends to be less useful than the correlation length, as it can be *significantly* influenced by the size of the area sampled. That is to say one can typically choose a sample area small enough to give *S*_*2D*_ *≅ 1* (perfect net order) or an area large enough to give *S*_*2D*_ *≅ 0* (no net order). The code may, however, be adapted to set an angle where a particular direction is induced *via* processes such as directional annealing[81] or graphoepitaxy; in such cases, *S*_*2D*_ *= -0*.*5* is a possibility for samples where the line orientation is orthogonal to the desired orientation.[2]

Finally, this skeleton-based approach facilitates generation of pseudo-coloured orientation maps, as in Fig 12, which also avoid grain-edge averaging problems exhibited with other methods.[82] Such images may assist researchers in qualitatively grasping the orientational ordering in their system. Such visual checks, can provide researchers with an accessible means of confirming numeric results, as it allows for a qualitative, direct measure of grain size on the image.

#### Stage 8: Output and confirmation images

**Fig 13:**
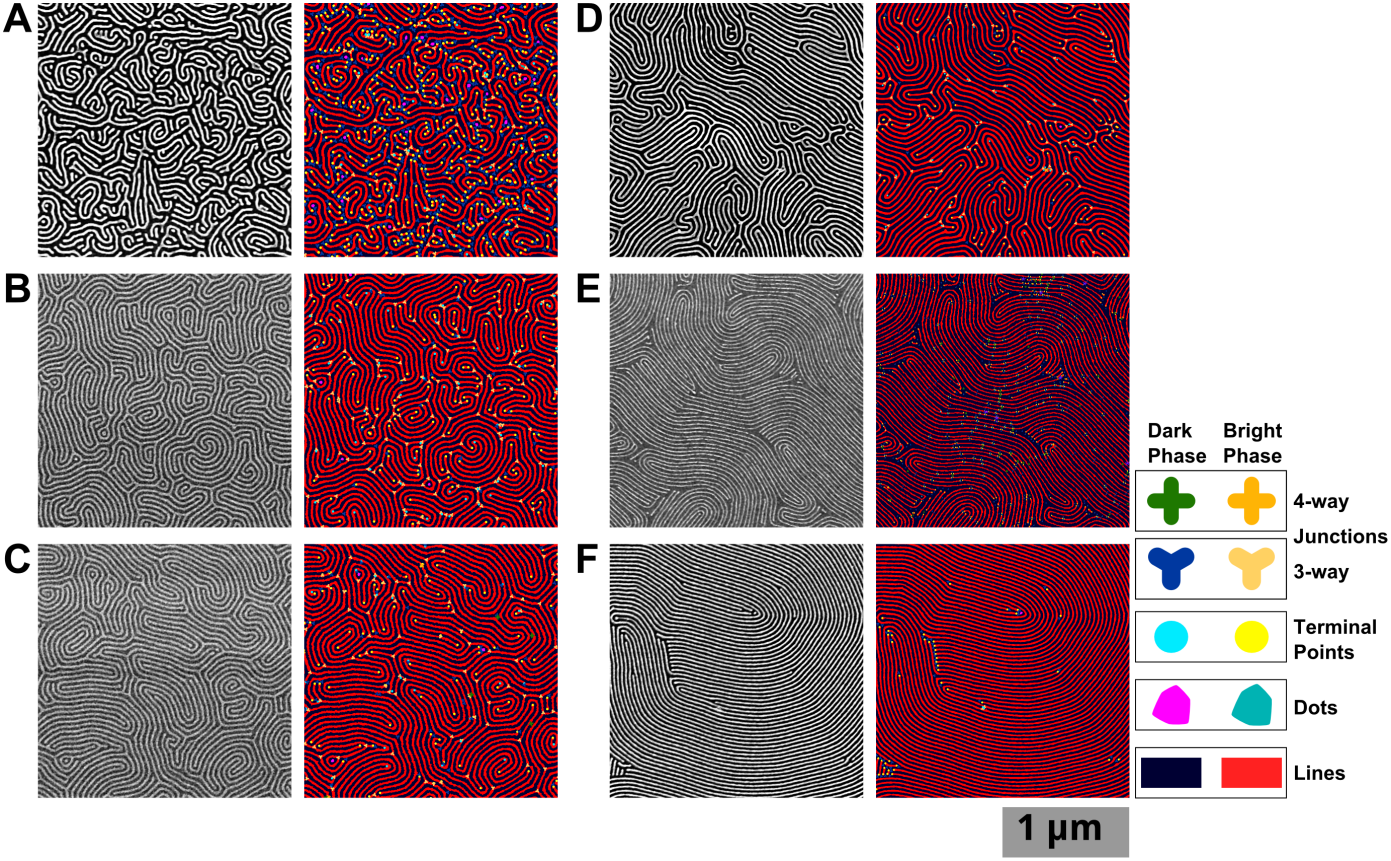
Original and defect analysis images for six SEM images of metallized PS-*b*-P2VP (50k-*b*-16.5k, 44k-*b*-18.5k, and 32.5k-*b*-12k) patterns with different degrees of thermal annealing. SEM images on left, and confirmation images with defects identified shown right. These images are spectacular only upon a close-up. Each image is shown cropped here to ~2 μm wide. The scale bar is 1 μm. (See S5 Fig for full images). The labels (A-F) show correspondence to the same processed images in Fig 12.

Finally, as a result of these considerations, we seek to provide self-assurance and quality control by creating confirmation images, wherein features described numerically are mapped onto real images to provide visual feedback of the accuracy of the measurement, as shown in Fig 13, which shows the defects found alongside the associated SEM images. This step is ultimately the means to determine whether the defects identified are (1) a true representation of the pattern and (2) are in the correct location. Such images of pattern orientation, line roughness, defects, and thresholding provide visual confirmation that all stages of the analysis proceeded correctly. Specifically, one can check simultaneously whether the thresholding, connectivity, grooming, and defect identification have all functioned as expected.

**Fig 14:**
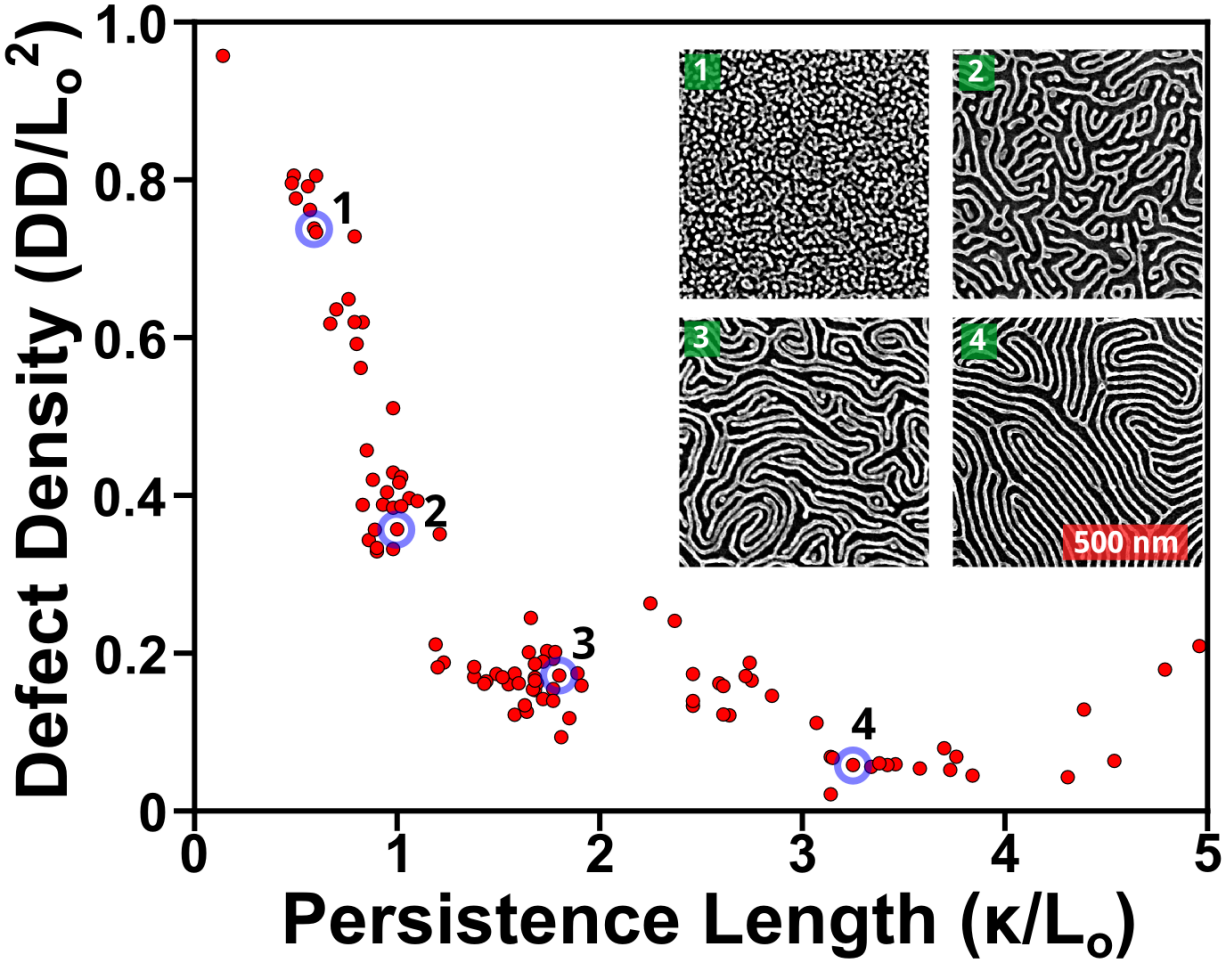
Relationship between correlation lengths, plotted here as persistence length (κ /L_o_) versus the defect density, normalized per unit period squared. Based on data for a variety of annealed, neat and blended, cylinder-forming, PS-*b*-P2VP polymer thin films of a variety of molecular weights including blends, using images of the metallized P2VP domains on Si substrates. This enables direct comparison between different polymers, which result in patterns with different periodicities. Defects initially show a dramatic decrease, as structures move away from dot arrays, for which the normalized, defect metric would be ~1. Inset displays four representative images. Respectively, their periods are 32, 43, 36, & 37 nm; their correlation lengths are 19, 43, 65, & 121 nm; their defect densities are 744, 195, 134, & 42 defect pairs·μm^-2^.

**Fig 15:**
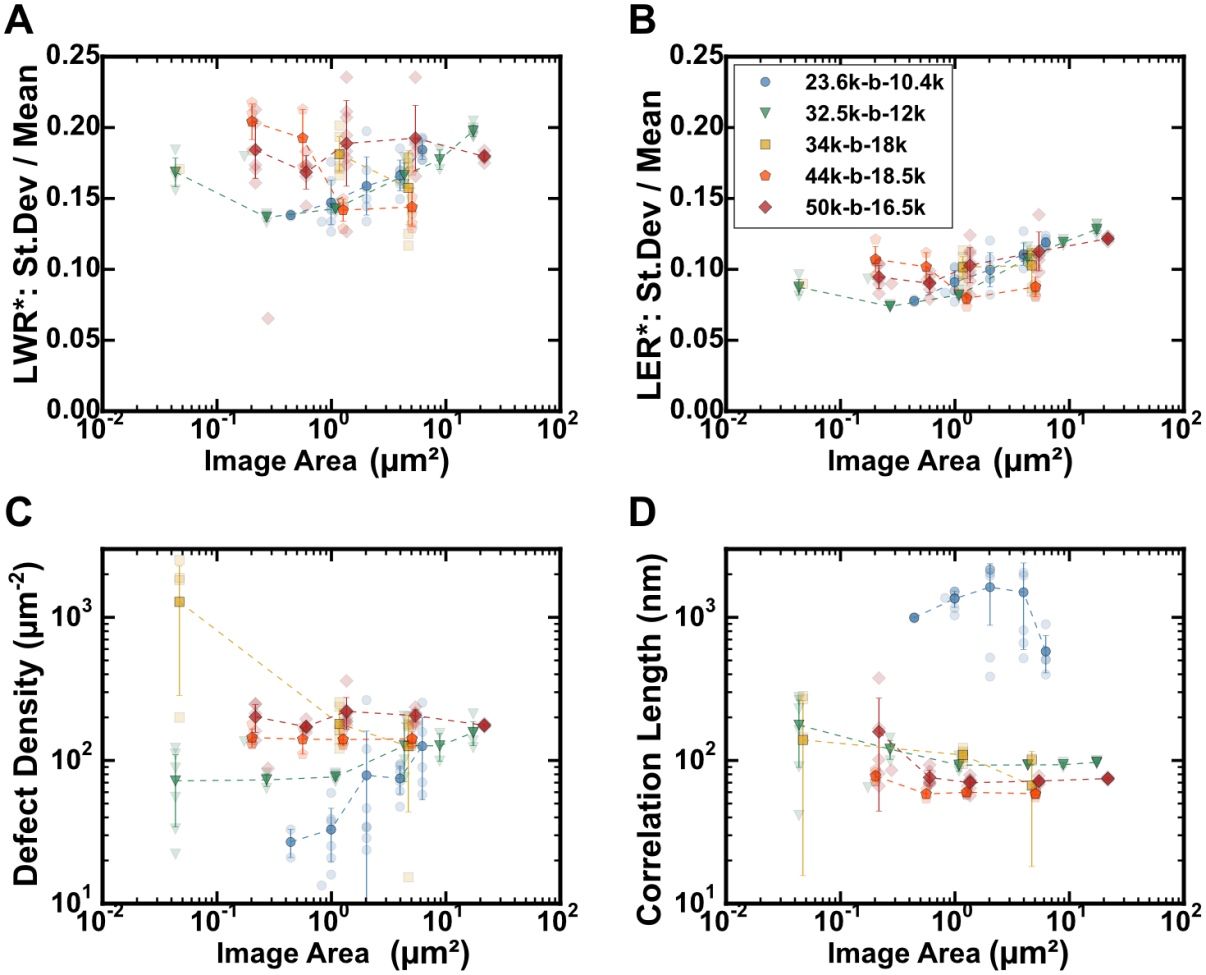
Data showing effect of sampling area and resolution for BCP pattern metrics: LWR, LER, defect density, and correlation length. All images had areas of 1280 x 896 pixels, taken with different magnification factors. Five cylinder-forming PS-b-P2VP block copolymers, each identically treated, were imaged: PS(23.6k)-b-P2VP(10.4k) [blue circles], PS(32.5k)-b-P2VP(12k) [green triangles], PS(34k)-b-P2VP(18k) [yellow squares], PS(44k)-b-P2VP(18.5k) [orange pentagons], and PS(50k)-b-P2VP(16.5k) [red diamonds]. Average values are indicated by dark markers and standard deviation error bars; data from individual images are shown with light markers. A. Standard deviation for line-width (LWR, 1σ) divided by the line-width for various resolutions and plotted as a function of real image area, μm^2^. B. Standard deviation for line edge position (LER, 1σ) divided by the line-width for various resolutions and plotted as a function of real image area, μm^2^. C. Defect pair density as a function of real image area, μm^2^. D. Correlation length measured as a function of real image area, μm^2^; see also S9 Fig, which plots the correlation length as a function of the number of grains measured.

**Fig 16:**
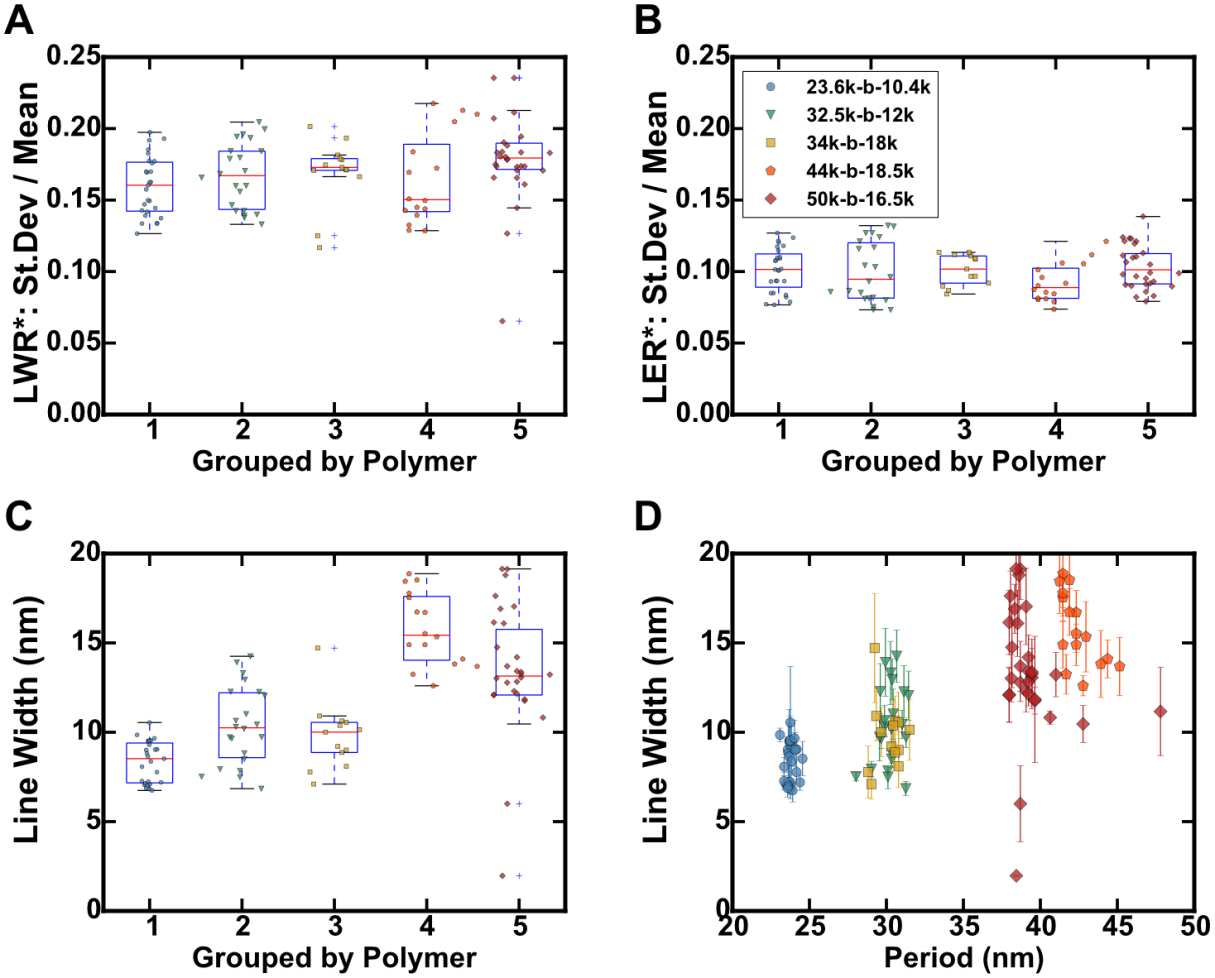
Boxplots of calculated BCP pattern metrics for SEM images with various resolutions for 5 cylinder-forming PS-b-P2VP block copolymers, each identically treated. Data from all resolutions shown. Data from individual images are shown with dark markers. A. Boxplot of the standard deviation for line-width (LWR, 1σ) divided by the line-width for various resolutions, grouped by polymer. B. Boxplot of the standard deviation for line edge position (LER, 1σ) divided by the line-width for various resolutions, grouped by polymer. C. Boxplot of measured line-widths for polymer groups by polymer. D. Same data, plotted as a function of BCP period (nm), all resolutions included; the error bars are standard deviations for the line-widths from measuring the lines separately.

Such visual feedback also lets researchers, particularly those presently involved in synthetic work, to tangibly grasp the important aspects of the pattern quality. By encoding the information spatially with colours and shapes rather than relying purely on the abstraction of defect densities and correlation lengths, ADAblock’s visual feedback can function as a guiding indicator for selection of optimum structures and conditions. The data output, both numerical and visual, make it possible to engage in exploratory data analysis[83] to discover new trends, motifs, and outliers in the data available, as demonstrated in Fig 14 and later in Figs 15 and 16.

### Application of ADAblock

In order to demonstrate the utility and versatility of this application, two different scenarios and questions are posed. First, what is the effect of image resolution, and the area sampled, on the measured defect densities, LER, and other parameters for patterns derived from self-assembled BCP thin films? Secondly, what can we learn from investigating the data provided by these samples, by examining the relationships between different features, to identify features that warrant further investigation—and what does this suggest about the resulting properties of a self-assembled BCP film?

#### Effect of Resolution and Sampling Area

When measuring defect densities, correlation lengths, LER, and LWR, the area sampled and the resolution can potentially affect the measured results. Ideally, for any measurement, the effect of sample size must be analyzed and understood in order to obtain reliable results. To develop a general sense of how this and different polymer sizes are affected in the analysis, we annealed 5 different polymer types, each with approximately ideal thicknesses, for 20 minutes at 200°C and imaged the resulting metallized patterns at different magnifications.

The effect of resolution in the LWR measurements in Fig 15A appears to be minimal, although there is a slight downward trend with increasing resolution (smaller image area) for the two smallest polymers, where the LWR (1σ) values decrease from 0.18 to 0.14. The increase in LWR is primarily observed for those samples with the smallest period, which would likely be on account of pixelation of the lines, as suggested by S8 Fig. A confounding effect may also result from the decreased length of line sampled for images of higher resolution. LER data, on the other hand, shows a more consistent trend of decreasing LER with increasing resolution in Fig 15B. LER is likely more affected by pixelation due to the inability of the skeletonization process to precisely locate the line center, in particular when line-widths are a small, even number of pixels. In contrast, the line-width is not strongly constrained by the determination of the line centre. The magnitude of the decrease (-0.03 to -0.05 pixels) here is still small, given that image area changes by a factor of up to 100.

Sampling effects can be observed in the measurement of defect pair density at various resolutions in Fig 15C. High resolution images, depending on the distribution of defects, can completely avoid defects or oversample them. Here the smallest BCP (23.6k-b-10.4k) is most affected, due to having a larger grain size. The same effect can be observed for correlation length measurements in Fig 15D, although this affects all of the polymers. In order for the correlation length measurement to be meaningful, the measured value should be significantly shorter than the dimensions of the image. The decrease in the average measured correlation length as a function of the image area suggests that one may be able to estimate the true value based on the size of the image. The plot of 23.6k-*b*-10.4k is particularly telling because it shows the effect of sampling within a single grain or few grains (at low resolution) and the sudden decrease once more grains become involved. The limitation of large grains may be partly avoided by using automated data collection, combined image stitching, which has been demonstrated to be effective for imaging large areas with electron microscopy,[84,85] however as ordering approaches perfection, grain sizes become infinite,[86] and the correlation function will approach unity.

#### Feature Relationships

In order to derive lessons from the data, we undertake a form of exploratory data analysis to chart the relationships of different parameters observed. In particular, whether parameters such as LER and LWR are independent of the feature size, and how line-widths, polymers, and periods have a simple relationship.

Taking all of the data (across resolutions) for each polymer, we note that as a proportion of the line-widths, the standard deviations in the edge position (LER, 1σ) and line-width (LWR, 1σ) stay constant, about 10% and 16% respectively, indicating that the LER and LWR scale with the line-width dimension of the polymer, as shown in Fig 16A and 16B. The set point may be a property of a given BCP’s Flory-Huggins parameter, indicating a higher χ required. However we must caution that other factors, such as the processing, metallization, plasma treatment, and lack of alignment are convoluted with the roughness inherent to the polymer, preventing a direct conclusion. However this method should enable comparison between polymer templates and patterns translated from the BCP *via* etching or other means. The values observed here would however all exceed LWR targets set by the ITRS for LWR (3σ) of less than 6%: 1.1 nm for patterns with 18 nm feature size; or <0.6 nm for patterns with 10 nm feature size.[22] For aligned patterns, solvent annealed with water as a co-solvent, we have observed significantly better LER and LWR values.[29] We hypothesize that it may be the result of the water selectively partitioning inside of the P2VP block during annealing, resulting in a higher effective χ, leading to a smoother interface than we attain here with thermal annealing.

Line-width in Fig 16C and 16D shows the expected relationship of being proportionate to the period, although there does appear to be a greater spread in the width of lines than in the FFT-measured periods. This is likely an effect of thresholding, which needs to be done relative to each image. It may be possible for a specific polymer or a series of images to constrain the threshold, as a fraction of area, in order to obtain a narrower distribution of line-widths.

#### Limitations of the code

As with any programmed analysis, there are drawbacks and trade-offs made in analysis to optimize for speed or accuracy. The approximations we implemented are one reason that necessitates a full sharing of the code. ImageJ’s macro language is interpreted, hence it is slower in processing compared to plugins or other compiled programs. It is, however, easily edited and modified, which enables adaptation where modification may be required. The code was written so that it can be operated in a batch mode to process a folder of images, meaning that a series of images can be processed overnight, or while attending to other tasks. It should be cautioned that in the present state, as ADAblock continues to be developed, the code may produce a reproducible error for ~4% of images at present. Further refinement should reduce this error rate, but at present may limit a series from being completed. With manual intervention, however, the image can be skipped, or the settings modified, and the queue re-continued.

Typically an image with dimensions of 1280 x 896 pixels (the default of our SEM, for example) requires ~7 minutes to process when run on the standard personal computers that we used for testing. Higher pixel-resolution (*e*.*g*. 2560 x 1792) images require more time to process, roughly in proportion to the number of pixels. Given the automated nature of the program, it’s possible to run a queue of images overnight, rendering the increased processing time irrelevant.

In addition to images showing the locations of defects, the code saves several check images to act as references to help determine whether any errors have taken place or other undesirable operations. Consequently, ~ 16 MB is recorded to the disk for each image processed, as presently conFigd, although non-graphic data only accounts for less than 300 kB. (See S1 Instructions for a list of files output by the program.)

## Conclusions

We have developed a facile, automated, and reliable analysis for striped patterns derived from the self-assembly of BCP thin films, that integrates both conventional and newly developed techniques. This analysis is done in order to quantify defects and their types using a skeletonization-based method; to measure line-edge roughness; and to calculate Herman’s order parameter and the correlation length in a novel fashion, based upon the skeletonized structure. Moreover, the skeletonized structure provides information about the connectivity of patterns. We expect that this will be of use to others carrying out annealing studies and preliminary characterizations of novel self-assembling polymeric materials. Finally, for 5 block copolymers of similar composition, we have found the metallized patterns to have LER and LWR in roughly constant proportion to the line-width.

Ultimately, no one measurement provides a “complete description” of pattern quality; typically they are complementary. Hence this work represents an attempt to broaden the scope of analysis and to make tools which may not be readily accessible to all. Additionally having shared protocols, or at least protocols derived from a common origin, we might be able to standardize a broad toolset, providing consistent analysis *via* fully shared code.[87,88] We hope this aids comparisons between polymers, between papers, and between scientists seeking to understand the characteristics of block copolymers, and in addressing the numerous critical issues associated with block copolymer lithography.[89]

## Materials and Methods

PS-*b*-P2VP block copolymers were obtained from Polymer Source Inc., QC, in weight-averaged molecular weights of 23.6k-*b*-10.4k, 32.5k-*b*-12k, 34k-*b*-18k, 44k-*b*-18.5k, and 50k-*b*-16.5k and all with polydispersity below 1.1. Toluene was purchased from Fisher Scientific; concentrated H_2_SO_4_ from Caledon Laboratories; 30% H_2_O_2_(aq) from Sigma-Aldrich; and Na_2_PtCl_4_·xH_2_O from Strem Chemicals. Silicon wafers were obtained from University Wafer.

### Substrate Preparation

100 mm diameter, single-side polished silicon wafers were diced into squares with dimensions 1.0 cm x 1.0 cm. Prior to cleaning, substrates were scribed, on the unpolished side, with a diamond-tip, to mark the identity of each substrate as part of a set of 10. The samples were then immersed in methanol and sonicated for 15 minutes in glass beakers. Next, after rinsing each substrate square in a series of beakers filled with 18.2 MΩ∙cm water, the substrates were placed polished-side-up in PTFE beakers, and immersed in 6.0 mL of concentrated H_2_SO_4_, to which was added 2.0 mL of 30% H_2_O_2_, before placing the beaker to stand in an 80°C hot water bath for 20 minutes. The piranha solution was then decanted to a glass flask to cool prior to neutralization.

Following several rinses with water, the substrates were immersed in aqueous 1% NH_4_OH solution for 5 minutes to remove any surface sulfonate groups, prior to a final decant and replacement of the solution with 18 MΩ∙cm water. Typically samples were stored immersed in water with the top sealed with paraffin wax.

### Solutions & Spin Coating

Immediately prior to spin coating, each wafer was dried under a nitrogen stream. Once dry, the sample was analyzed using fixed-angle, single-wavelength ellipsometry (632.8 nm) to determine the thickness of the thermal oxide at the center; typically 2 nm. Spin coating was carried out under argon or nitrogen gas. Each substrate’s polished side was evenly coated with 10 μL of 10–15 g/L BCP solution; any bubbles were manually removed; then the substrates were spun for up to 15 s, between 3000 rpm and 4000 rpm, with an initial acceleration of 1500 rpm/s. Following this, the film was reanalyzed by ellipsometry, prior to quartering the sample and annealing.

### Annealing

Thermal annealing was carried out in ambient atmosphere on a hotplate covered with a thin aluminum sheet. Temperature was monitored directly at the wafer using an OSENSA fiber-optic fluorescence-based temperature probe. For the thickness measurements and for the comparison of the 5 polymers, the substrates were annealed for 20 minutes at 200°C.

### Metallization

A solution of 20 mM Na_2_PtCl_4_ in 0.9 M HCl_(aq)_ was used for metallizing PS-*b*-P2VP samples. Samples were submerged for at least 2–3 hours prior to removal and rinsing with 18.2 MΩ∙cm water.

### Plasma processing

Following metallization, sample sets were placed together in a plasma chamber, and the chamber was evacuated to < 200 mTorr to remove contaminant gases or adsorbates. Finally, O_2_ gas was leaked into the chamber to a pressure of ~ 750 mTorr. The RF coils were then energized and a faint lavender-blue O_2_ plasma was maintained for ~ 60 s (depending on the film thickness) to etch the organic materials from the substrate. Finally, samples were imaged using a Hitachi S-4800 scanning electron microscope, sampling regions near the centre of each substrate.

### Computation

For image analysis, ImageJ,[52] version 1.49 and above, was used. It is freely available at http://imagej.nih.gov/ij/. The code for performing the analyses is available on our institutional repository; updated versions will be available on GitHub. Python scripts used in preparing the data shown here are also available to assist with processing and plotting output from multiple runs. They are available under an MIT license, allowing users to freely copy, redistribute, and modify the code.

## Supporting Information

S1 Appendix Calculations of the geometry of line patterns. (PDF)

S1 Fig Median filtering and Gaussian filtering used to reduce noise. (A) Sample image with blue dotted line of (B) intensity profile. The left series (C,E,G) shows the effect of a median filter; the right series (D,F,H) shows the effect of a Gaussian filter. (C,D) Each filter applied at a 5-pixel radius. (E,F) Histograms of each filtered image. (G,H) The smoothing observed for filtering in a range of 0 to 10 pixels, shown in series: (H) Gaussian-filtered profile loses contrast more quickly than (G) the median-filtered profile.
(TIF)

S2 Fig Feret measurement for curved objects. (A) ImageJ can measure the calliper width of objects (shown by the arrows), however only using a MinFeret measurement, which is obtained using a rotating callipers method of rotation the objects perimeter to find the minimum height occupied by the selection. (B) For curved or bent line objects, this would result in a width greater than the linewidth, thus making this method inapplicable to highly disordered block copolymer systems.
(TIF)

S3 Fig Equivalency justification for determining the defect value for each disclination. (A) A dot can be thought of as being a junctionless line which has been reduced in length to its width. The +½ defect value associated with each of the two terminal points can be viewed as combining to give the +1 value of the dot. This can also be seen when the dots are paired with-½ defects. The 3-branch junction has a value of-½. Although 3-branch junctions are the most common, (C) 4-branch junctions (-1) and (C) even 5-branch junctions (-1½) can, on rare occasion, be observed. For each additional branch, the value decreases by ½; this can be viewed as being equivalent to sliding an additional branch from a 3-branch junction to increase the junction by 1 branch.
(TIF)

S4 Fig Rules regarding defects with regard to their location in the image frame. (A) Dots not touching the edge are counted in full. (B) Dot-sized features touching the edge are counted as half-dots, equivalent to terminal points. (C) For line features which lie on the edge, they do not count, as the terminal points are not within the frame of the image. (D) Dots touching two edges do not count, as these can be considered equivalent to lines touching two edges as in (E) and (F), which do not contribute to the defectivity of the image. (G) and (H) Only the terminal points and junctions within the frame of the image are counted for any given lines.
(TIF)

S5 Fig Full, processed images shown cropped in Figs 10 and 12 of the paper. Full resolution images with labels (A-F) corresponding to those in Figs 10 and 12 of the paper.
(TIF)

S6 Fig Striking image showing blended overlay of analysis with SEM image. Original SEM image from panel (D) of Figs 10 and 12, and S5 Fig, blended with overlays of defect analysis on the left and orientation map on the right, all at full resolution.
(TIF)

S7 Fig Pairing of defects and defect unit cells. Negative defects are noted with magenta dots, while negative dots are identified with yellow dots. The defect unit cells, shown in yellow, are drawn to indicate the parts of the defect required for the defect pairs to be part of an otherwise homogeneous, ideal pattern. (A) Region with an ideal pattern. (B) Region of dot pattern, which represents the maximum possible defect density for a stripe pattern made from a misoriented cylindrical BCP. (C) An isolated junction, which cannot be part of a unit cell on its own. (D) An isolated terminal point, also not part of a unit cell on its own. (E) H-junctions formed either from a line break or (F) from a bridging of two lines. (G)/(H) Junctions created by two adjacent lines coming into contact. (H) is similar to (H), except that the defect at the centre is 4-connected. (I) Paired dislocations. (J) Paired disclination pairs, where positive and negative components are in the same phase.
(TIF)

S8 Fig Pattern period in pixels as a function of image area. Using images with constant dimensions (1280 pixels wide by 896 pixels high), and various image resolutions (500k magnification to 20k magnification), pattern periods were determined automatically from azimuthally averaged fast Fourier transform images. The relationship, within these constraints, for each of the 5 polymers is shown, with individual measurements shown as lighter markers; the dark markers are the averaged data. Dashed lines function as a guide for each set.
(TIF)

S9 Fig Grain count affects estimates of correlation length. Using data from Fig 15D, the number of grains in each image is approximated by dividing the image area by the grain size, taking the average grain area to be a circle with radius equal to the correlation length. Correlation lengths are also normalized, dividing the measured correlation length for each image by an estimate of the “true” correlation length (listed on each subplot) which would be measured for an image of the entire surface. A vertical dashed line on each image at 10 grains serves a reference point. The following values were used as estimates of the “true” correlation length for each image: PS(23.6k)-b-P2VP(10.4k): 500 nm, PS(32.5k)-b-P2VP(12k): 95 nm, PS(34k)-b-P2VP(18k): 101 nm, PS(44k)-b-P2VP(18.5k): 58 nm, and PS(50k)-b-P2VP(16.5k): 72 nm.
(TIF)

S1 Instructions Use of ADAblock. (PDF)

## References

[1] Stoykovich MP, Müller M, Kim SO, Solak HH, Edwards EW, Pablo JJ de, et al Directed Assembly of Block Copolymer Blends into Nonregular Device-Oriented Structures. Science. 2005;308: 1442–1446. 10.1126/science.1111041 15933196

[2] Jung YS, Ross CA. Orientation-Controlled Self-Assembled Nanolithography Using a Polystyrene−Polydimethylsiloxane Block Copolymer. Nano Lett. 2007;7: 2046–2050. 10.1021/nl070924l 17570733

[3] Black CT, Guarini KW, Milkove KR, Baker SM, Russell TP, Tuominen MT. Integration of self-assembled diblock copolymers for semiconductor capacitor fabrication. Applied Physics Letters. 2001;79: 409–411. 10.1063/1.1383805

[4] Tavakkoli K.G. A, Gotrik KW, Hannon AF, Alexander-Katz A, Ross CA, Berggren KK. Templating Three-Dimensional Self-Assembled Structures in Bilayer Block Copolymer Films. Science. 2012;336: 1294–1298. 10.1126/science.1218437 22679094

[5] Ruiz R, Dobisz E, Albrecht TR. Rectangular Patterns Using Block Copolymer Directed Assembly for High Bit Aspect Ratio Patterned Media. ACS Nano. 2011;5: 79–84. 10.1021/nn101561p 21182251

[6] Yang X, Wan L, Xiao S, Xu Y, Weller DK. Directed Block Copolymer Assembly versus Electron Beam Lithography for Bit-Patterned Media with Areal Density of 1 Terabit/inch^2^ and Beyond. ACS Nano. 2009;3: 1844–1858. 10.1021/nn900073r 19572736

[7] Ruiz R, Kang H, Detcheverry FA, Dobisz E, Kercher DS, Albrecht TR, et al Density Multiplication and Improved Lithography by Directed Block Copolymer Assembly. Science. 2008;321: 936–939. 10.1126/science.1157626 18703735

[8] Kim E, Ahn H, Park S, Lee H, Lee M, Lee S, et al Directed Assembly of High Molecular Weight Block Copolymers: Highly Ordered Line Patterns of Perpendicularly Oriented Lamellae with Large Periods. ACS Nano. 2013;7: 1952–1960. 10.1021/nn3051264 23441640

[9] Mistark PA, Park S, Yalcin SE, Lee DH, Yavuzcetin O, Tuominen MT, et al Block-Copolymer-Based Plasmonic Nanostructures. ACS Nano. 2009;3: 3987–3992. 10.1021/nn901245w 19947582

[10] Deeg JA, Louban I, Aydin D, Selhuber-Unkel C, Kessler H, Spatz JP. Impact of Local versus Global Ligand Density on Cellular Adhesion. Nano Lett. 2011;11: 1469–1476. 10.1021/nl104079r 21425841 PMC3806292

[11] Kruss S, Wolfram T, Martin R, Neubauer S, Kessler H, Spatz JP. Stimulation of Cell Adhesion at Nanostructured Teflon Interfaces. Adv Mater. 2010;22: 5499–5506. 10.1002/adma.201003055 20972983

[12] Adutler-Lieber S, Zaretsky I, Platzman I, Deeg J, Friedman N, Spatz JP, et al Engineering of synthetic cellular microenvironments: Implications for immunity. Journal of Autoimmunity. 2014;54: 100–111. 10.1016/j.jaut.2014.05.003 24951031

[13] Park S, Kim B, Wang J-Y, Russell TP. Fabrication of Highly Ordered Silicon Oxide Dots and Stripes from Block Copolymer Thin Films. Adv Mater. 2008;20: 681–685. 10.1002/adma.200701997

[14] Chai J, Wang D, Fan X, Buriak JM. Assembly of aligned linear metallic patterns on silicon. Nat Nano. 2007;2: 500–506. 10.1038/nnano.2007.227 18654348

[15] Hong SW, Gu W, Huh J, Sveinbjornsson BR, Jeong G, Grubbs RH, et al On the Self-Assembly of Brush Block Copolymers in Thin Films. ACS Nano. 2013;7: 9684–9692. 10.1021/nn402639g 24156297

[16] Cushen JD, Otsuka I, Bates CM, Halila S, Fort S, Rochas C, et al Oligosaccharide/Silicon-Containing Block Copolymers with 5 nm Features for Lithographic Applications. ACS Nano. 2012;6: 3424–3433. 10.1021/nn300459r 22456229

[17] Bates CM, Seshimo T, Maher MJ, Durand WJ, Cushen JD, Dean LM, et al Polarity-Switching Top Coats Enable Orientation of Sub–10-nm Block Copolymer Domains. Science. 2012;338: 775–779. 10.1126/science.1226046 23139327

[18] In I, La Y-H, Park S-M, Nealey PF, Gopalan P. Side-Chain-Grafted Random Copolymer Brushes as Neutral Surfaces for Controlling the Orientation of Block Copolymer Microdomains in Thin Films. Langmuir. 2006;22: 7855–7860. 10.1021/la060748g 16922574

[19] Mansky P, Russell TP, Hawker CJ, Mays J, Cook DC, Satija SK. Interfacial Segregation in Disordered Block Copolymers: Effect of Tunable Surface Potentials. Phys Rev Lett. 1997;79: 237–240. 10.1103/PhysRevLett.79.237

[20] Park M, Harrison C, Chaikin PM, Register RA, Adamson DH. Block Copolymer Lithography: Periodic Arrays of ~10^11^ Holes in 1 Square Centimeter. Science. 1997;276: 1401–1404. 10.1126/science.276.5317.1401

[21] Jeong CK, Baek KM, Niu S, Nam TW, Hur YH, Park DY, et al Topographically-Designed Triboelectric Nanogenerator via Block Copolymer Self-Assembly. Nano Lett. 2014;14: 7031–7038. 10.1021/nl503402c 25393064

[22] Emerging Research Materials (2013). Albany, NY: SEMATECH; 2014 Apr. Available: http://www.itrs.net

[23] Ouk Kim S, Solak HH, Stoykovich MP, Ferrier NJ, de Pablo JJ, Nealey PF. Epitaxial self-assembly of block copolymers on lithographically defined nanopatterned substrates. Nature. 2003;424: 411–414. 10.1038/nature01775 12879065

[24] Stoykovich MP, Kang H, Daoulas KC, Liu G, Liu C-C, de Pablo JJ, et al Directed Self-Assembly of Block Copolymers for Nanolithography: Fabrication of Isolated Features and Essential Integrated Circuit Geometries. ACS Nano. 2007;1: 168–175. 10.1021/nn700164p 19206647

[25] Wu NLY, Harris KD, Buriak JM. Conversion of Bilayers of PS-b-PDMS Block Copolymer into Closely Packed, Aligned Silica Nanopatterns. ACS Nano. 2013;7: 5595–5606. 10.1021/nn401968t 23675942

[26] Bita I, Yang JKW, Jung YS, Ross CA, Thomas EL, Berggren KK. Graphoepitaxy of Self-Assembled Block Copolymers on Two-Dimensional Periodic Patterned Templates. Science. 2008;321: 939–943. 10.1126/science.1159352 18703736

[27] Ross CA, Jung YS, Chuang VP, Ilievski F, Yang JKW, Bita I, et al Si-containing block copolymers for self-assembled nanolithography. J Vac Sci Technol, B. 2008;26: 2489–2494. 10.1116/1.2981079

[28] Zhang X, Harris KD, Wu NLY, Murphy JN, Buriak JM. Fast Assembly of Ordered Block Copolymer Nanostructures through Microwave Annealing. ACS Nano. 2010;4: 7021–7029. 10.1021/nn102387c 20964379

[29] Wu NLY, Zhang X, Murphy JN, Chai J, Harris KD, Buriak JM. Density Doubling of Block Copolymer Templated Features. Nano Lett. 2012;12: 264–268. 10.1021/nl203488a 22168820

[30] Kim Y, Pyun J, Fréchet JMJ, Hawker CJ, Frank CW. The Dramatic Effect of Architecture on the Self-Assembly of Block Copolymers at Interfaces. Langmuir. 2005;21: 10444–10458. 10.1021/la047122f 16262305

[31] Poelma JE, Ono K, Miyajima D, Aida T, Satoh K, Hawker CJ. Cyclic Block Copolymers for Controlling Feature Sizes in Block Copolymer Lithography. ACS Nano. 2012;6: 10845–10854. 10.1021/nn304217y 23194415

[32] Hawker CJ, Bosman AW, Harth E. New Polymer Synthesis by Nitroxide Mediated Living Radical Polymerizations. Chem Rev. 2001;101: 3661–3688. 10.1021/cr990119u 11740918

[33] Hawker CJ, Wooley KL. The Convergence of Synthetic Organic and Polymer Chemistries. Science. 2005;309: 1200–1205. 10.1126/science.1109778 16109874

[34] Hawker CJ. “Living” Free Radical Polymerization: A Unique Technique for the Preparation of Controlled Macromolecular Architectures. Acc Chem Res. 1997;30: 373–382. 10.1021/ar960248m

[35] Bates FS, Fredrickson GH. Block Copolymers—Designer Soft Materials. Physics Today. 1999;52: 32–38. 10.1063/1.882522

[36] Gopalan P, Yang S. Etch-Resistant Block Copolymers. Material Matters. 2006;1: 6.

[37] Fukukawa K, Zhu L, Gopalan P, Ueda M, Yang S. Synthesis and Characterization of Silicon-Containing Block Copolymers from Nitroxide-Mediated Living Free Radical Polymerization. Macromolecules. 2005;38: 263–270. 10.1021/ma049217u

[38] Beardsley TM, Matsen MW. Monte Carlo Phase Diagram for a Polydisperse Diblock Copolymer Melt. Macromolecules. 2011;44: 6209–6219. 10.1021/ma200966a

[39] Lynd NA, Hillmyer MA. Influence of Polydispersity on the Self-Assembly of Diblock Copolymers. Macromolecules. 2005;38: 8803–8810. 10.1021/ma051025r

[40] Lynd NA, Hillmyer MA. Effects of Polydispersity on the Order−Disorder Transition in Block Copolymer Melts. Macromolecules. 2007;40: 8050–8055. 10.1021/ma070962r

[41] Sweat DP, Kim M, Larson SR, Choi JW, Choo Y, Osuji CO, et al Rational Design of a Block Copolymer with a High Interaction Parameter. Macromolecules. 2014;47: 6687–6696. 10.1021/ma501597g

[42] Nagpal U, Müller M, Nealey PF, de Pablo JJ. Free Energy of Defects in Ordered Assemblies of Block Copolymer Domains. ACS Macro Lett. 2012;1: 418–422. 10.1021/mz200245s 35578514

[43] Horvat A, Sevink GJA, Zvelindovsky AV, Krekhov A, Tsarkova L. Specific Features of Defect Structure and Dynamics in the Cylinder Phase of Block Copolymers. ACS Nano. 2008;2: 1143–1152. 10.1021/nn800181m 19206332

[44] Takahashi H, Laachi N, Delaney KT, Hur S-M, Weinheimer CJ, Shykind D, et al Defectivity in Laterally Confined Lamella-Forming Diblock Copolymers: Thermodynamic and Kinetic Aspects. Macromolecules. 2012;45: 6253–6265. 10.1021/ma300993x

[45] Harrison CK. Block copolymer microdomains in thin films [Internet]. Ph.D., Princeton University. 1999. Available: http://search.proquest.com/docview/304542401/abstract

[46] Harrison C, Adamson DH, Cheng Z, Sebastian JM, Sethuraman S, Huse DA, et al Mechanisms of Ordering in Striped Patterns. Science. 2000;290: 1558–1560. 10.1126/science.290.5496.1558 11090350

[47] Harrison C, Chaikin PM, Huse DA, Register RA, Adamson DH, Daniel A, et al Reducing Substrate Pinning of Block Copolymer Microdomains with a Buffer Layer of Polymer Brushes. Macromolecules. 2000;33: 857–865. 10.1021/ma991551g

[48] Qiang Z, Zhang Y, Groff JA, Cavicchi KA, Vogt BD. A generalized method for alignment of block copolymer films: solvent vapor annealing with soft shear. Soft Matter. 2014;10: 6068–6076. 10.1039/C4SM00875H 25004006

[49] Tseng Y-C, Darling SB. Block Copolymer Nanostructures for Technology. Polymers. 2010;2: 470–489. 10.3390/polym2040470

[50] Zhang X, Murphy JN, Wu NLY, Harris KD, Buriak JM. Rapid Assembly of Nanolines with Precisely Controlled Spacing from Binary Blends of Block Copolymers. Macromolecules. 2011;44: 9752–9757. 10.1021/ma202064t

[51] Jin C, Murphy JN, Harris KD, Buriak JM. Deconvoluting the Mechanism of Microwave Annealing of Block Copolymer Thin Films. ACS Nano. 2014;8: 3979–3991. 10.1021/nn5009098 24655292

[52] Rasband WS. ImageJ. Bethesda, Maryland, USA: U. S. National Institutes of Health; 1997 Available: http://imagej.nih.gov/ij/

[53] Schneider CA, Rasband WS, Eliceiri KW. NIH Image to ImageJ: 25 years of image analysis. Nat Meth. 2012;9: 671–675. 10.1038/nmeth.2089 PMC5554542 22930834

[54] Bencher C, Yi H, Zhou J, Cai M, Smith J, Miao L, et al Directed self-assembly defectivity assessment. Part II. 2012 p. 83230N–83230N. 10.1117/12.917993

[55] Otsu Nobuyuki. A Threshold Selection Method from Gray-Level Histograms. IEEE Transactions on Systems, Man and Cybernetics. 1979;9: 62–66. 10.1109/TSMC.1979.4310076

[56] Landini G. Auto Local Threshold. 2013. Available: https://github.com/fiji/Auto_Threshold/tree/master/src/main/java/fiji/threshold

[57] Doube M, Kłosowski MM, Arganda-Carreras I, Cordelières FP, Dougherty RP, Jackson JS, et al BoneJ: Free and extensible bone image analysis in ImageJ. Bone. 2010;47: 1076–1079. 10.1016/j.bone.2010.08.023 20817052 PMC3193171

[58] Zhang TY, Suen CY. A Fast Parallel Algorithm for Thinning Digital Patterns. Commun ACM. 1984;27: 236–239. 10.1145/357994.358023

[59] Rehse S, Mecke K, Magerle R. Characterization of the dynamics of block copolymer microdomains with local morphological measures. Phys Rev E. 2008;77: 051805 10.1103/PhysRevE.77.051805 18643095

[60] Scherdel S, Schoberth HG, Magerle R. Visualizing the dynamics of complex spatial networks in structured fluids. The Journal of Chemical Physics. 2007;127: 014903 10.1063/1.2747598 17627365

[61] Vigild ME, Almdal K, Mortensen K, Hamley IW, Fairclough JPA, Ryan AJ. Transformations to and from the Gyroid Phase in a Diblock Copolymer. Macromolecules. 1998;31: 5702–5716. 10.1021/ma9716746

[62] Campbell IP, He C, Stoykovich MP. Topologically Distinct Lamellar Block Copolymer Morphologies Formed by Solvent and Thermal Annealing. ACS Macro Lett. 2013;2: 918–923. 10.1021/mz400269k 35607014

[63] Abukhdeir NM, Rey AD. Defect kinetics and dynamics of pattern coarsening in a two-dimensional smectic-A system. New J Phys. 2008;10: 063025 10.1088/1367-2630/10/6/063025

[64] Kléman M. Points, lines, and walls: in liquid crystals, magnetic systems, and various ordered media. Chichester; New York: J. Wiley; 1983.

[65] Windows Minesweeper. MinesweeperWiki. 2013. Available: http://www.minesweeper.info/wiki/Windows_Minesweeper

[66] Campbell IP, Hirokawa S, Stoykovich MP. Processing Approaches for the Defect Engineering of Lamellar-Forming Block Copolymers in Thin Films. Macromolecules. 2013;46: 9599–9608. 10.1021/ma401704m

[67] Kim H-W, Lee J-Y, Shin J, Woo S-G, Cho H-K, Moon J-T. Experimental investigation of the impact of LWR on sub-100-nm device performance. IEEE Transactions on Electron Devices. 2004;51: 1984–1988. 10.1109/TED.2004.839115

[68] Asenov A, Kaya S, Brown AR. Intrinsic parameter fluctuations in decananometer MOSFETs introduced by gate line edge roughness. IEEE Transactions on Electron Devices. 2003;50: 1254–1260. 10.1109/TED.2003.813457

[69] Stucchi M, Bamal M, Maex K. Impact of line-edge roughness on resistance and capacitance of scaled interconnects. Microelectronic Engineering. 2007;84: 2733–2737. 10.1016/j.mee.2007.05.038

[70] Steinhögl W, Schindler G, Steinlesberger G, Traving M, Engelhardt M. Impact of line edge roughness on the resistivity of nanometer-scale interconnects. Microelectronic Engineering. 2004;76: 126–130. 10.1016/j.mee.2004.07.005

[71] Patrone PN, Gallatin GM. Modeling line-edge roughness in lamellar block copolymer systems. 2012 p. 83232Q–83232Q–9. 10.1117/12.918038

[72] Patrone PN, Gallatin GM. Modeling Line Edge Roughness in Templated, Lamellar Block Copolymer Systems. Macromolecules. 2012;45: 9507–9516. 10.1021/ma301421j

[73] Peters AJ, Lawson RA, Ludovice PJ, Henderson CL. Effects of block copolymer polydispersity and χN on pattern line edge roughness and line width roughness from directed self-assembly of diblock copolymers Proc SPIE 8680. San Jose, California, USA: SPIE; 2013 pp. 868020–1–868020–8. 10.1117/12.2021443

[74] Emerging Research Materials (2011).Albany, NY: SEMATECH; 2011 Dec. Available: http://www.itrs.net

[75] Wu NL-Y. Self-Assembly of Block Copolymers for Nanopatterning. Thesis, University of Alberta. 2014. Available: http://hdl.handle.net/10402/era.37764

[76] Isawa M, Sakai K, Rincon Delgadillo PA, Gronheid R, Yoshida H. Line edge roughness measurement technique for fingerprint pattern in block copolymer thin film Proc SPIE 8681. San Jose, California, USA: SPIE; 2013 pp. 868114–868114–6. 10.1117/12.2010915

[77] templatetypedef. Answer to: Determining if two line segments intersect? In: Stack Overflow. 12 Feb 2011. Available: http://stackoverflow.com/a/4977569. Accessed 12 August 2014.

[78] Garetz BA, Balsara NP, Dai HJ, Wang Z, Newstein MC, Majumdar B. Orientation Correlations in Lamellar Block Copolymers. Macromolecules. 1996;29: 4675–4679. 10.1021/ma9600724

[79] Chang MY, Abuzaina FM, Kim WG, Gupton JP, Garetz BA, Newstein MC, et al Analysis of Grain Structure in Partially Ordered Block Copolymers by Depolarized Light Scattering and Transmission Electron Microscopy. Macromolecules. 2002;35: 4437–4447. 10.1021/ma0201025

[80] Hu X, Zhu Y, Gido SP, Russell TP, Iatrou H, Hadjichristidis N, et al The effect of molecular architecture on the grain growth kinetics of AnBn star block copolymers. Faraday Discuss. 2005;128: 103–112. 10.1039/B403881A 15658769

[81] Qiang Z, Zhang L, Stein GE, Cavicchi KA, Vogt BD. Unidirectional Alignment of Block Copolymer Films Induced by Expansion of a Permeable Elastomer during Solvent Vapor Annealing. Macromolecules. 2014;47: 1109–1116. 10.1021/ma402131j

[82] Berry BC, Bosse AW, Douglas JF, Jones RL, Karim A. Orientational Order in Block Copolymer Films Zone Annealed below the Order−Disorder Transition Temperature. Nano Lett. 2007;7: 2789–2794. 10.1021/nl071354s 17691851

[83] Brillinger DR. International Encyclopedia of Political Science Data Analysis, Exploratory. Thousand Oaks, CA: SAGE Publications, Inc.; 2011 pp. 531–538. Available: http://knowledge.sagepub.com/view/intlpoliticalscience/n128.xml

[84] Faas FGA, Avramut MC, Berg BM van den, Mommaas AM, Koster AJ, Ravelli RBG. Virtual nanoscopy: Generation of ultra-large high resolution electron microscopy maps. J Cell Biol. 2012;198: 457–469. 10.1083/jcb.201201140 22869601 PMC3413355

[85] Williams EH, Carpentier P, Misteli T. The JCB DataViewer scales up. J Cell Biol. 2012;198: 271–272. 10.1083/jcb.201207117 22869591 PMC3413368

[86] Majewski PW, Yager KG. Millisecond Ordering of Block Copolymer Films via Photothermal Gradients. ACS Nano. 2015;9: 3896–3906. 10.1021/nn5071827 25763534

[87] Ince DC, Hatton L, Graham-Cumming J. The case for open computer programs. Nature. 2012;482: 485–488. 10.1038/nature10836 22358837

[88] Niemeyer K. Nature Editorial: If you want reproducible science, the software needs to be open source. In: Ars Technica. 26 Feb 2012. Available: http://arstechnica.com/science/2012/02/science-code-should-be-open-source-according-to-editorial/. Accessed 26 February 2012.

[89] Bates CM, Maher MJ, Janes DW, Ellison CJ, Willson CG. Block Copolymer Lithography. Macromolecules. 2014;47: 2–12. 10.1021/ma401762n

