# Supplementary material for: Automated Defect and Correlation Length Analysis of Block Copolymer Thin Film Nanopatterns
Source: PLoS One. 2015 Jul 24;10(7):e0133088. doi: 10.1371/journal.pone.0133088 (PMC4514826; doi:10.1371/journal.pone.0133088)
Supplement: S1 Appendix — (PDF) [file pone.0133088.s001.pdf]

Note S1: Calculations on the Geometry of Line Patterns

One can imagine several different scenarios for the "tips" of lines, and how they would contribute to the overall relationship between line perimeter and line area. A structure consisting entirely of junctions—and analyzed as such, without breaking lines apart—would have junctions contributing a small area without any additional contribution to the perimeter. We can approximate this by junctions coming to meet in an equilateral triangle, as shown in the following figure:

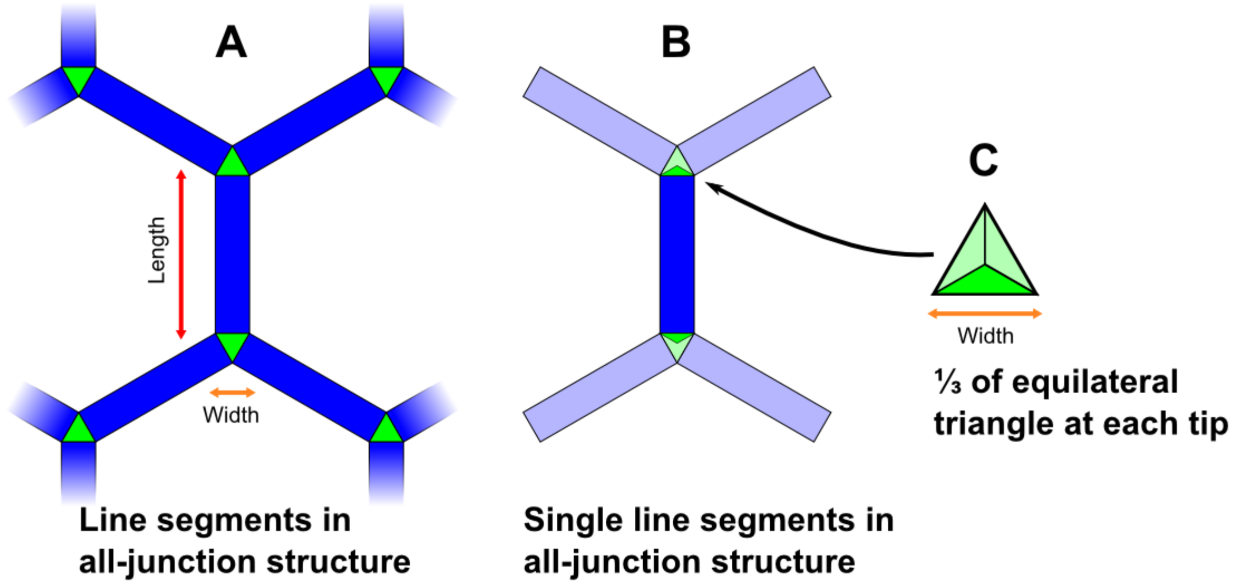

Figure S1.1. A. Shows part of a hypothetical infinite array of connected line segments; such lines could be parallel, however the hexagonal layout simplifies depiction. B. Shows an isolated line, with associated area from the triangular region at each junction. C. The area at each tip can be approximated as ⅓ of an equilateral triangle.

For this continuous network, we can calculate the area as a product of the length of each segment, multiplied by its width, plus two triangular pieces, each ⅓ of an equilateral triangle, for which the edge length is equal to the width:

$$A = Lw + 2\frac{A_\Delta}{3}$$

Substituting the area of an equilateral triangle for A_Δ, we have:

$$A = Lw + \frac{\sqrt{3}w^2}{6}$$

The perimeter is simply twice the length of the line segment, as the junctions contribute no area in this case:

$P = 2L$

Rearranging to make the area depend on perimeter, one obtains:

$$A(P) = \tfrac{1}{2}wP + \tfrac{\sqrt{3}}{6}w^2 \approx \tfrac{1}{2}wP + 0.289w^2$$

The slope remains ½w, however the intercept is $0.289w^2$, which for a line of width 18 nm, were it to only have junctions and arbitrary lengths, one would expect an intercept of +93.5 nm^2.

For samples with lines that terminate and do not possess junctions, the equation can be modified to presume a semi-circular tip at the end. As shown below:

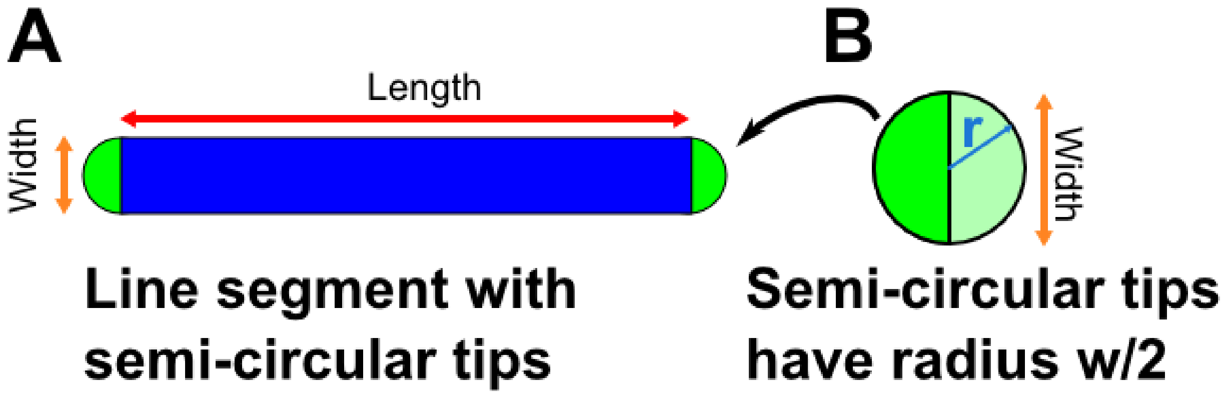

Figure S1.2. A. Lines without any junctions can be approximated as having semicircles for tips. B. These would correspond to circles with a radius half the width of the line.

From this we can contrive, using the usual equations for a circle, an equation for area,

$$A = Lw + A_{circle} = Lw + \pi r^2 = Lw + \tfrac{1}{4}\pi w^2$$

and similarly for the perimeter,

$$P = 2L + P_{circle} = 2L + \pi w$$

of the line segment.Converting the equation for area to a function of perimeter gives:

$$A = \left(\tfrac{P-\pi w}{2}\right)w + \tfrac{1}{4}\pi w^2 = \tfrac{1}{2}wP - \tfrac{1}{4}\pi w^2 \approx \tfrac{1}{2}wP - 0.785w^2$$

The intercept for a structure with semicircular tips would be $-0.785w^2$, which for a line of width 18 nm, would correspond to a value of -254.5 nm^2, for a collection of lines of constant width and tip structure, but arbitrary length.

Finally, perhaps the simplest case would be for the lines to have a simple rectangular structure with no tip-area that is unaccounted for by the length, as drawn:

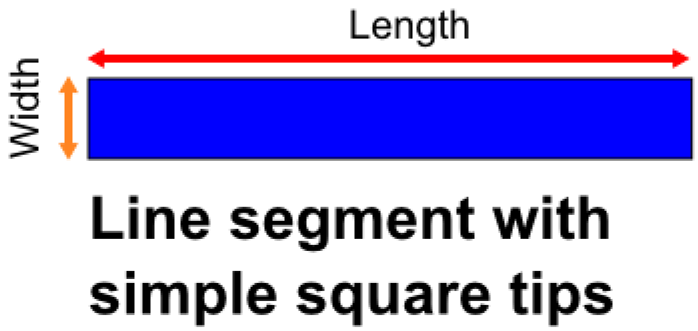

Figure S1.3. Depiction of a line segment with no junctions and regular, square tips.

The area would simply be the product of the length and the width,

$$A = Lw$$

while the perimeter would be twice the sum of the length and the width,

$$P = 2(L + w)$$

Rearranging to give the area as a function of perimeter:

$$A = \tfrac{1}{2}wP - w^2$$

The intercept would be $-w^2$, which for a 18 nm wide line would be -324 nm^2. Figure 3 provides examples for one such image, where the intercept, after removing junction-containing structures, is -104.4 nm^2, indicates that the semi-circular tips would be a better model, on average.

Furthermore, for such a sample image, the magnitude of the change makes for a scatter which is small compared to the typical particle areas: on a range of 5000 nm^2 to 30000 nm^2, ±300 nm^2 represents ± 0.06 % to ± 0.01 %.

Typically samples with moderate levels of defects will have a mixture of particles with varying numbers of junctions and terminal points. Larger particles tend to characteristically have a greater number of junctions; smaller particles fewer junctions. Additionally, particles in contact with the edge will tend to be smaller due to being cut out of the image frame and possibly an increased perimeter relative to area. Removal of junctions and parts of images in contact with the edge eliminates the varied “intercept” of individual points, pushing the data toward the same line (without junctions) as shown in the figure below. Consequently, the slope will

represent solely the width of the line; any component resulting from a scatter in the “intercept” is minimized.

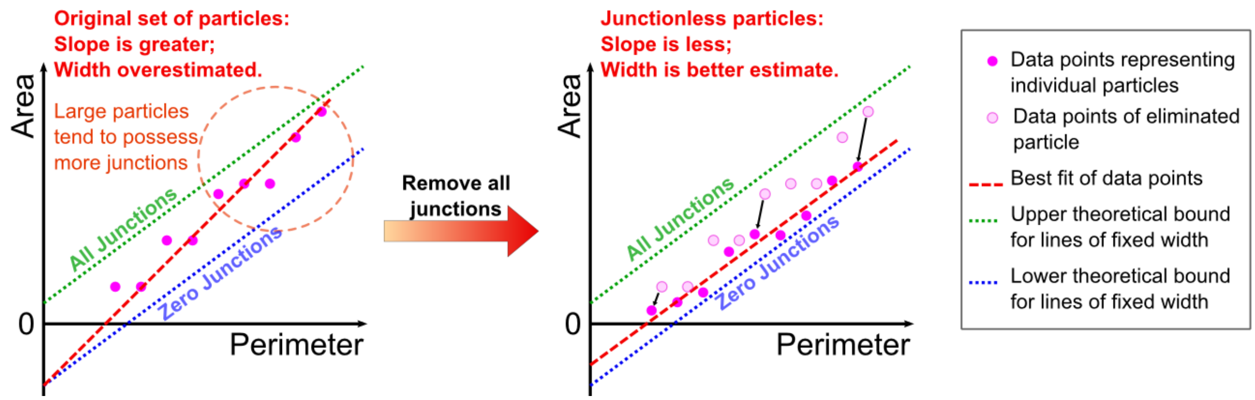

Figure S1.4. Schematic graphs depicting the effect of removing junctions and particles touching the edge of an image. On the left, larger particles tend to bias the slope higher, due to the increase in area for samples of an equivalent perimeter; overall there is greater scatter of the data points. On the right, removal of junctions results in a smaller scatter in the data points, and places them closer to the theoretical slope, as dependent on the width.
