# Supplementary figures and images for: Automated Defect and Correlation Length Analysis of Block Copolymer Thin Film Nanopatterns
Source: PLoS One. 2015 Jul 24;10(7):e0133088. doi: 10.1371/journal.pone.0133088 (PMC4514826; doi:10.1371/journal.pone.0133088)

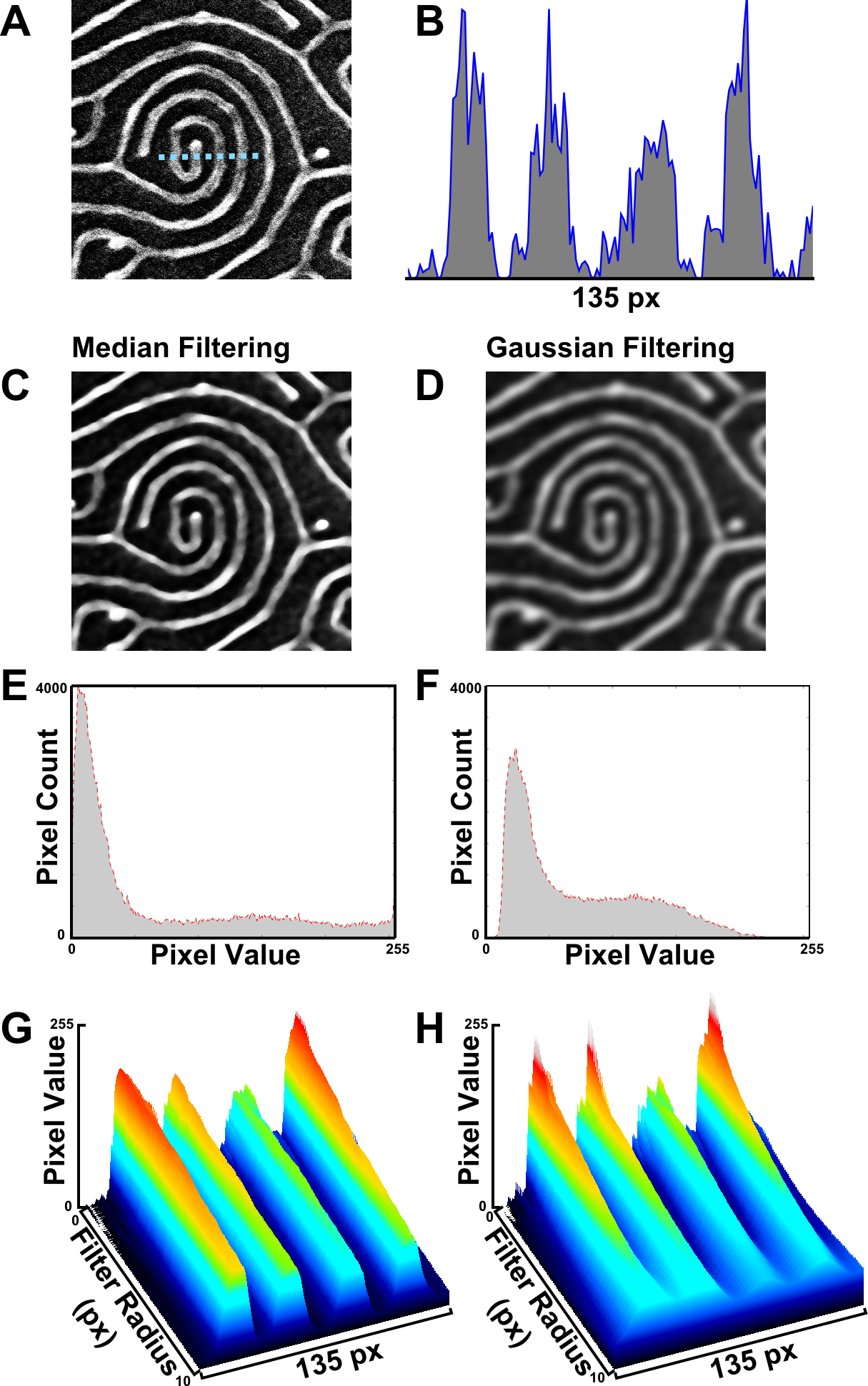

Supplement: S1 Fig — (A) Sample image with blue dotted line of (B) intensity profile. The left series (C,E,G) shows the effect of a median filter; the right series (D,F,H) shows the effect of a Gaussian filter. (C,D) Each filter applied at a 5-pixel radius. (E,F) Histograms of each filtered image. (G,H) The smoothing observed for filtering in a range of 0 to 10 pixels, shown in series: (H) Gaussian-filtered profile loses contrast more quickly than (G) the median-filtered profile. (TIF) [file pone.0133088.s002.tif]

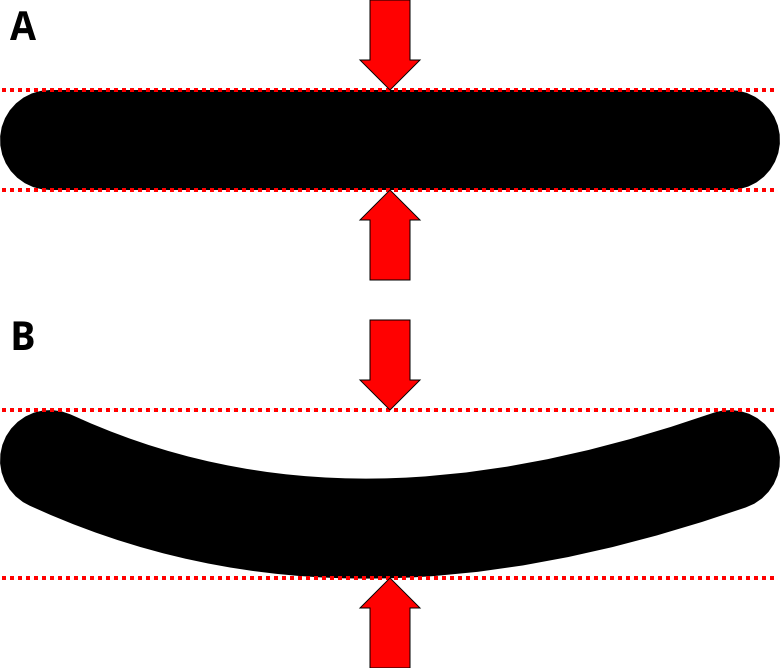

Supplement: S2 Fig — (A) ImageJ can measure the calliper width of objects (shown by the arrows), however only using a MinFeret measurement, which is obtained using a rotating callipers method of rotation the objects perimeter to find the minimum height occupied by the selection. (B) For curved or bent line objects, this would result in a width greater than the linewidth, thus making this method inapplicable to highly disordered block copolymer systems. (TIF) [file pone.0133088.s003.tif]

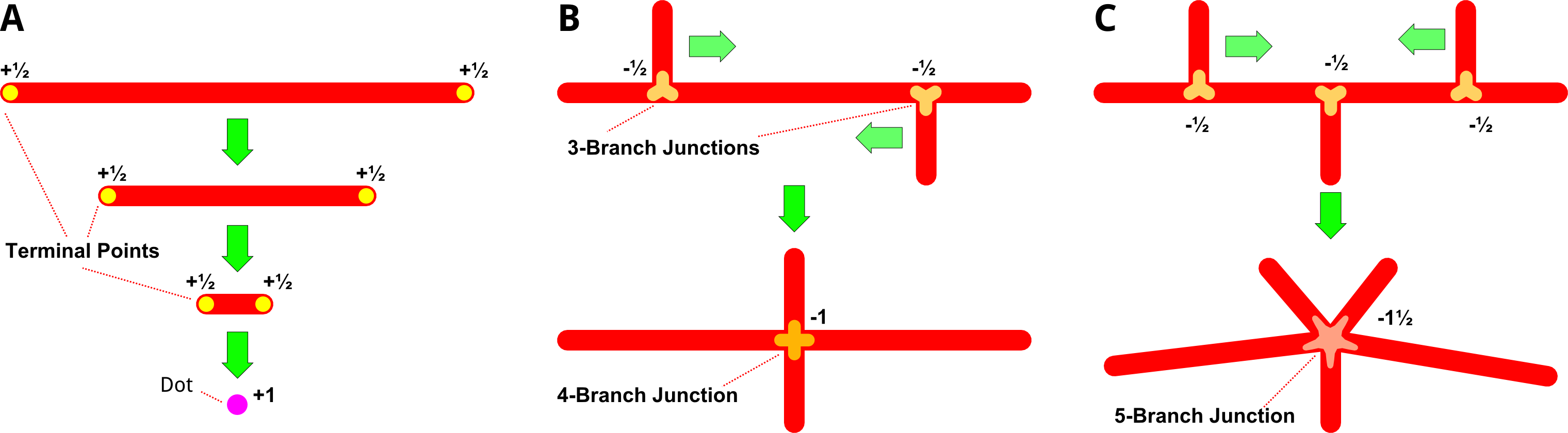

Supplement: S3 Fig — (A) A dot can be thought of as being a junctionless line which has been reduced in length to its width. The +½ defect value associated with each of the two terminal points can be viewed as combining to give the +1 value of the dot. This can also be seen when the dots are paired with-½ defects. The 3-branch junction has a value of-½. Although 3-branch junctions are the most common, (C) 4-branch junctions (-1) and (C) even 5-branch junctions (-1½) can, on rare occasion, be observed. For each additional branch, the value decreases by ½; this can be viewed as being equivalent to sliding an additional branch from a 3-branch junction to increase the junction by 1 branch. (TIF) [file pone.0133088.s004.tif]

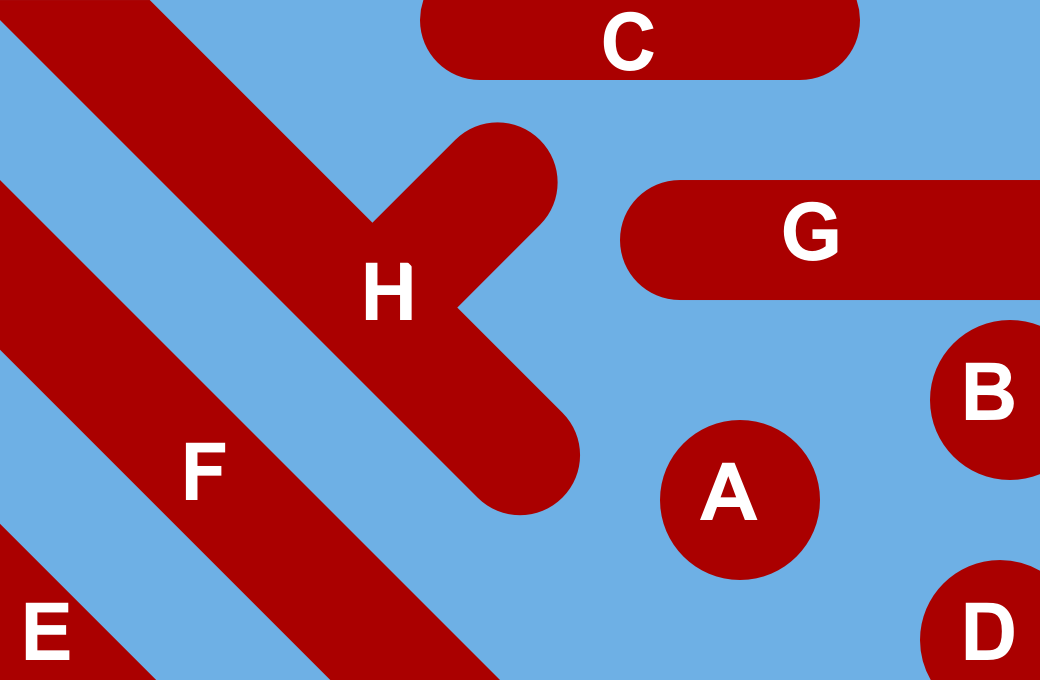

Supplement: S4 Fig — (A) Dots not touching the edge are counted in full. (B) Dot-sized features touching the edge are counted as half-dots, equivalent to terminal points. (C) For line features which lie on the edge, they do not count, as the terminal points are not within the frame of the image. (D) Dots touching two edges do not count, as these can be considered equivalent to lines touching two edges as in (E) and (F), which do not contribute to the defectivity of the image. (G) and (H) Only the terminal points and junctions within the frame of the image are counted for any given lines. (TIF) [file pone.0133088.s005.tif]

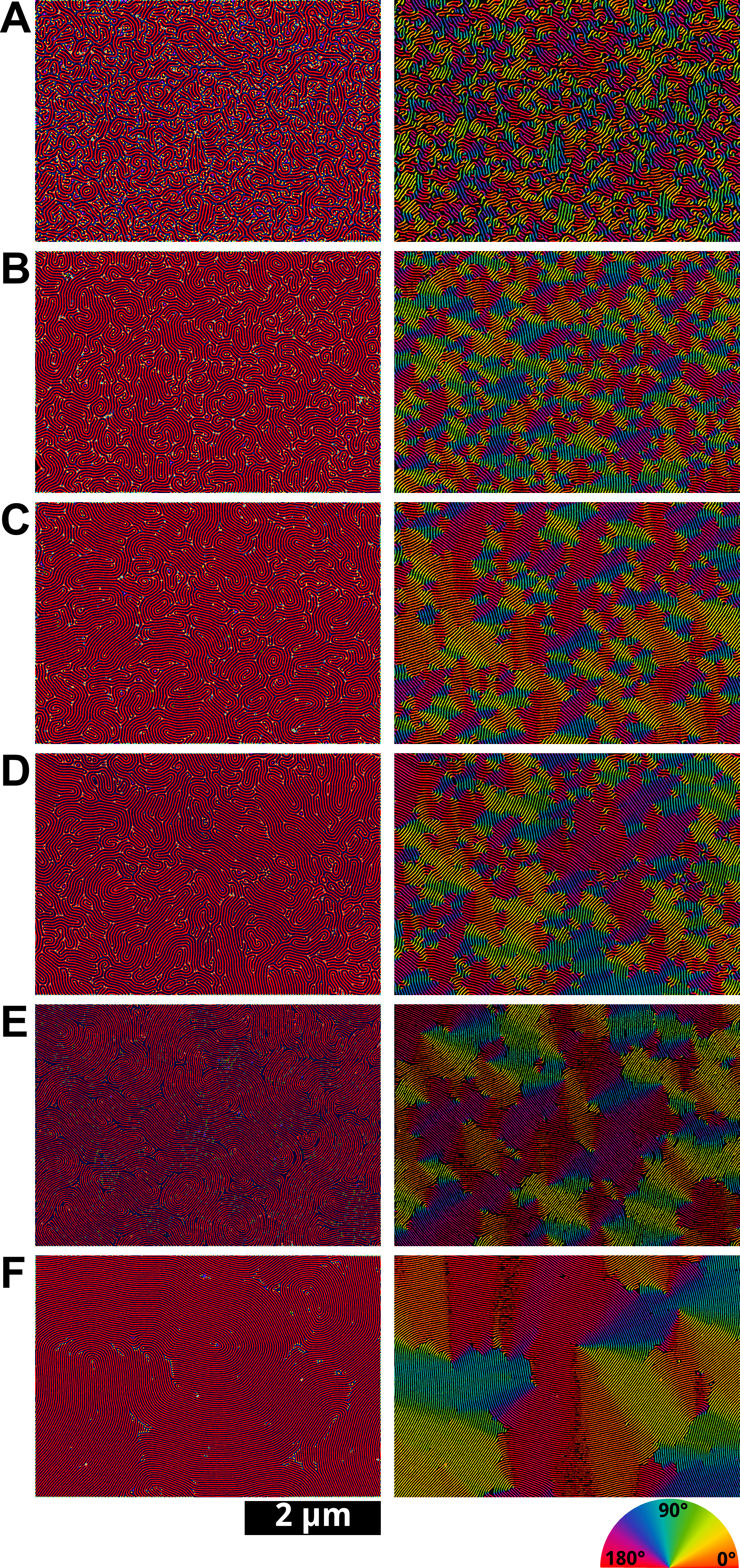

Supplement: S5 Fig — Full resolution images with labels (A-F) corresponding to those in Figs 10 and 12 of the paper. (TIF) [file pone.0133088.s006.tif]

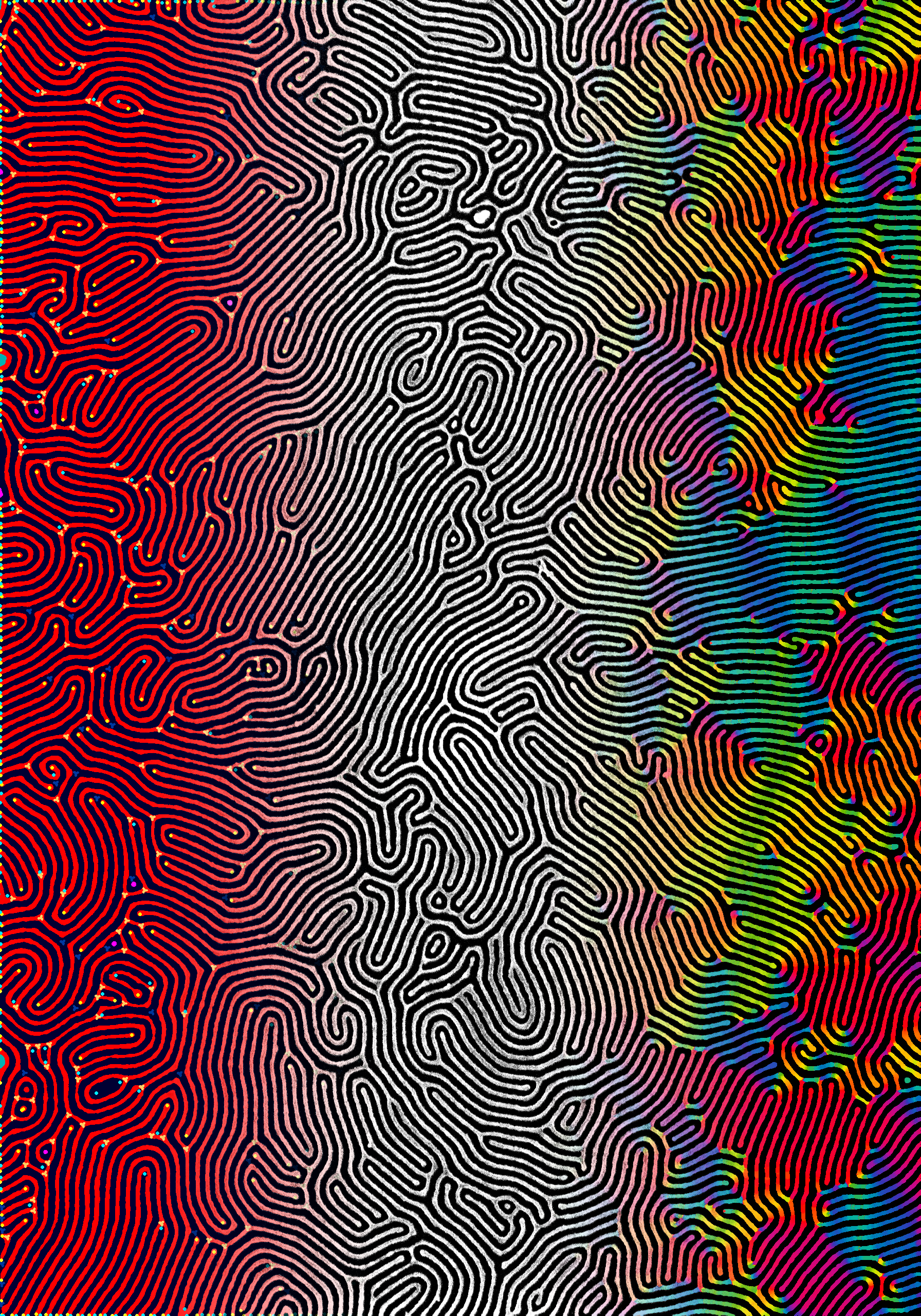

Supplement: S6 Fig — Original SEM image from panel (D) of Figs 10 and 12, and S5 Fig, blended with overlays of defect analysis on the left and orientation map on the right, all at full resolution. (TIF) [file pone.0133088.s007.tif]

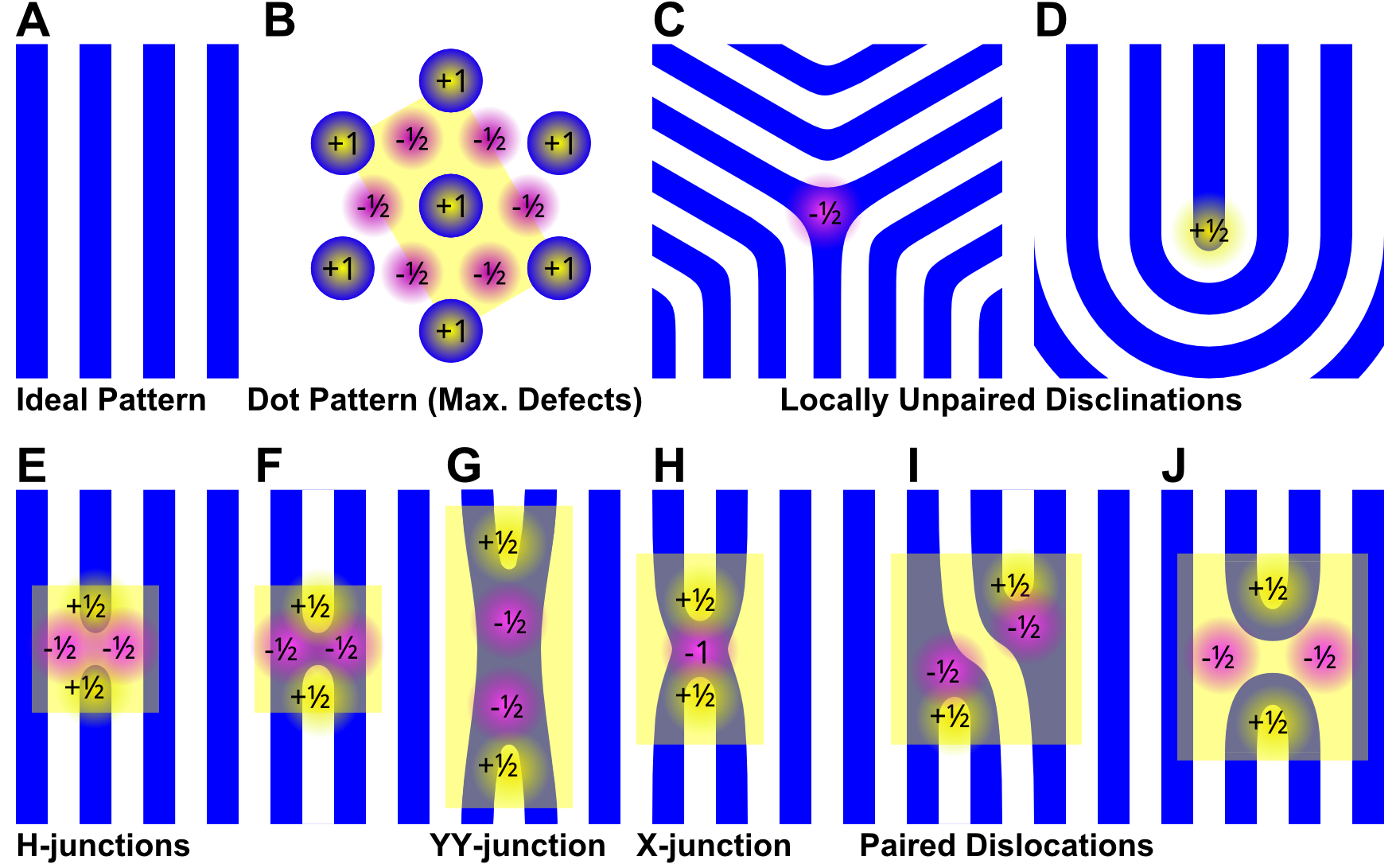

Supplement: S7 Fig — Negative defects are noted with magenta dots, while negative dots are identified with yellow dots. The defect unit cells, shown in yellow, are drawn to indicate the parts of the defect required for the defect pairs to be part of an otherwise homogeneous, ideal pattern. (A) Region with an ideal pattern. (B) Region of dot pattern, which represents the maximum possible defect density for a stripe pattern made from a misoriented cylindrical BCP. (C) An isolated junction, which cannot be part of a unit cell on its own. (D) An isolated terminal point, also not part of a unit cell on its own. (E) H-junctions formed either from a line break or (F) from a bridging of two lines. (G)/(H) Junctions created by two adjacent lines coming into contact. (H) is similar to (H), except that the defect at the centre is 4-connected. (I) Paired dislocations. (J) Paired disclination pairs, where positive and negative components are in the same phase. (TIF) [file pone.0133088.s008.tif]

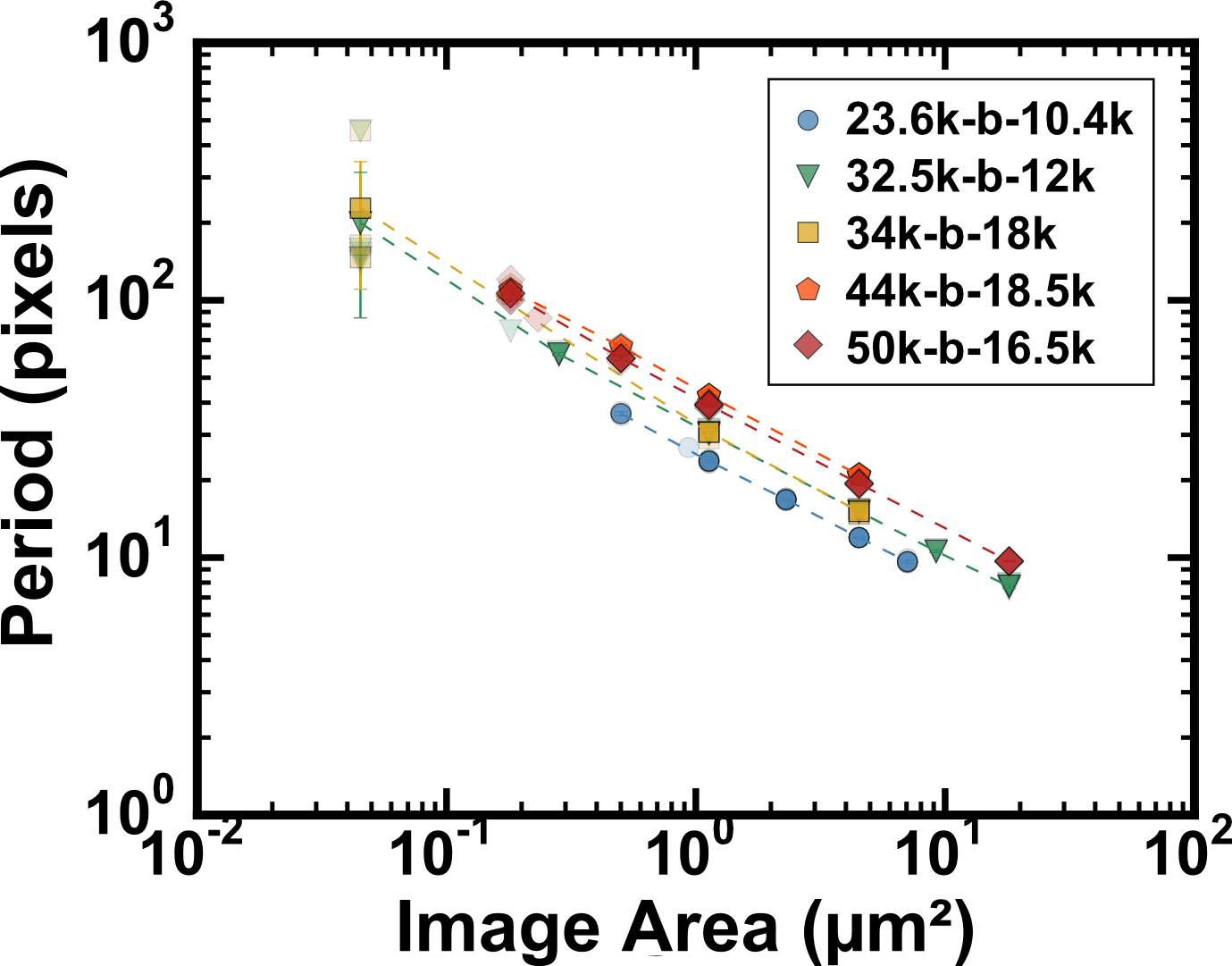

Supplement: S8 Fig — Using images with constant dimensions (1280 pixels wide by 896 pixels high), and various image resolutions (500k magnification to 20k magnification), pattern periods were determined automatically from azimuthally averaged fast Fourier transform images. The relationship, within these constraints, for each of the 5 polymers is shown, with individual measurements shown as lighter markers; the dark markers are the averaged data. Dashed lines function as a guide for each set. (TIF) [file pone.0133088.s009.tif]

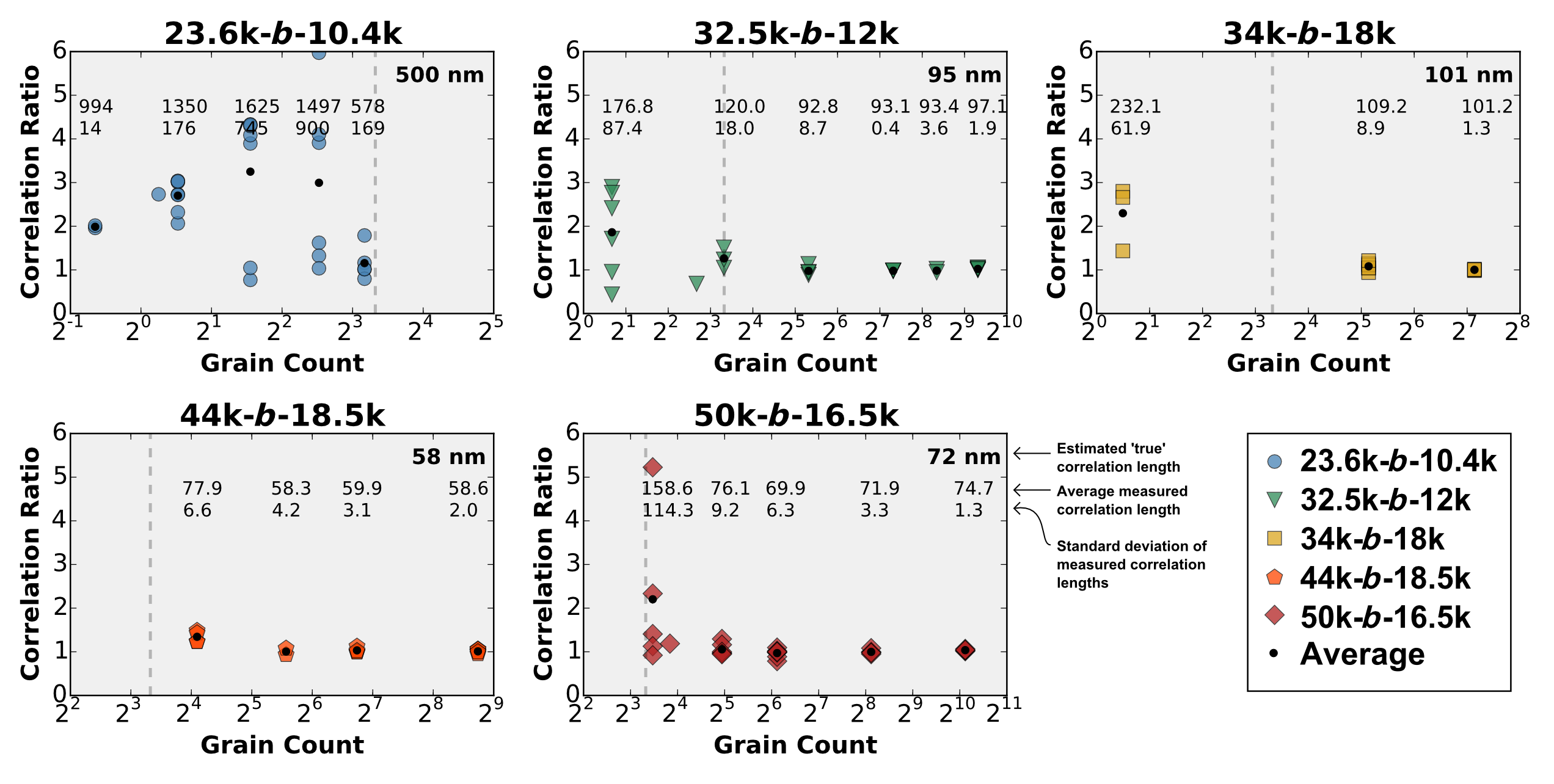

Supplement: S9 Fig — Using data from Fig 15D, the number of grains in each image is approximated by dividing the image area by the grain size, taking the average grain area to be a circle with radius equal to the correlation length. Correlation lengths are also normalized, dividing the measured correlation length for each image by an estimate of the “true” correlation length (listed on each subplot) which would be measured for an image of the entire surface. A vertical dashed line on each image at 10 grains serves a reference point. The following values were used as estimates of the “true” correlation length for each image: PS(23.6k)-b-P2VP(10.4k): 500 nm, PS(32.5k)-b-P2VP(12k): 95 nm, PS(34k)-b-P2VP(18k): 101 nm, PS(44k)-b-P2VP(18.5k): 58 nm, and PS(50k)-b-P2VP(16.5k): 72 nm. (TIF) [file pone.0133088.s010.tif]
