# Supplementary material for: Automated Defect and Correlation Length Analysis of Block Copolymer Thin Film Nanopatterns
Source: PLoS One. 2015 Jul 24;10(7):e0133088. doi: 10.1371/journal.pone.0133088 (PMC4514826; doi:10.1371/journal.pone.0133088)
Supplement: S1 Instructions — (PDF) [file pone.0133088.s011.pdf]

Instructions S1: Use of ADAblock

Set-up ImageJ

1. Install ImageJ. The current version is 1.49c. Previous versions may be inadequate and it may not be immediately apparent.

- If you do not have ImageJ, it can be downloaded, free of charge, from: http://imagej.nih.gov/ij/
- If ImageJ is already installed, check the current version: *Help > About ImageJ…*
- If ImageJ is not the current version: *Help > Update ImageJ…* and choose the most recent version from the list.

2. Install additional plugins. They are typically provided as ***.jar** or ***.class** files, which need to be placed in the ***~/ImageJ/plugins*** folder, followed by restarting ImageJ.

- **Auto Local Threshold**, by Gabriel Landini: http://fiji.sc/Auto_Local_Threshold (last updated November 2013)

3. Increase the memory allocation for ImageJ. This can be modified at: *Edit > Options > Memory & Threads…* Although not necessarily required, the settings used in the course of our data processing were:

- Maximum memory: 2000 MB
- Parallel threads: 6
- Keep multiple undo buffers: False
- Run garbage collector on status bar click: True

Run the Algorithm:

4. Open the algorithm: Either 1) Navigate within ImageJ: *Plugins > Macros > Edit…* , then find the **IntegratedAlgorithm.ijm** file to open it, or 2) Drag the **IntegratedAlgorithm.ijm** file to the ImageJ status bar (at the bottom of the window) and release. The message, ***<<Drag and Drop>>*** will appear, and the macro will open in a text editing window.

5. Run the macro: From the editing window, select *Macro > Run Macro*, or press [Ctrl + R].

Alternatively, the macro can be run without opening it in the editor, by navigating within ImageJ: *Plugins > Macros > Run…* , then find the **IntegratedAlgorithm.ijm** file to open & run.

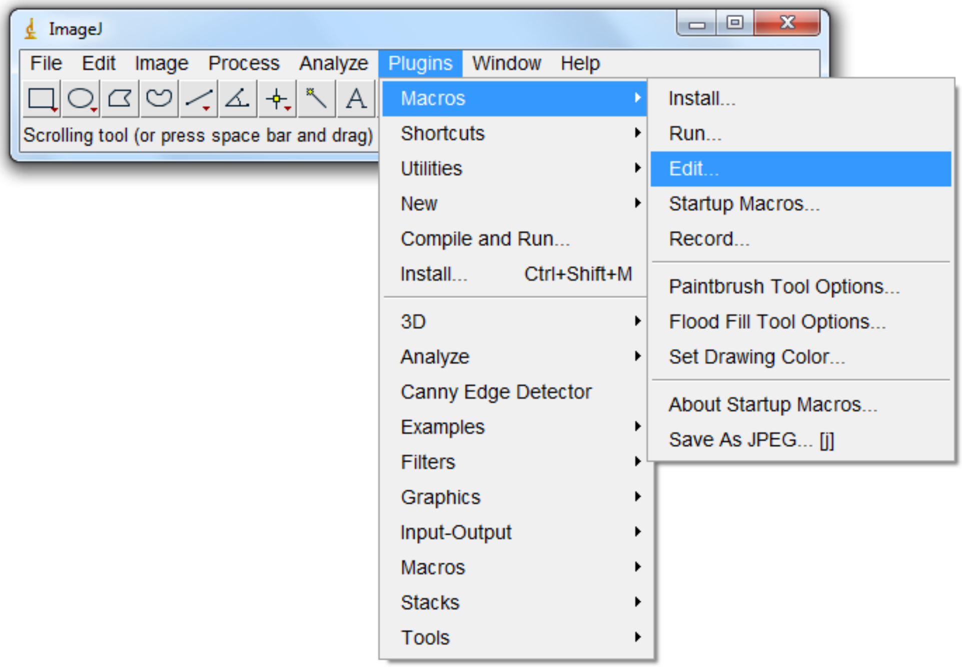

Screenshot: *Plugins > Macros > Edit…*

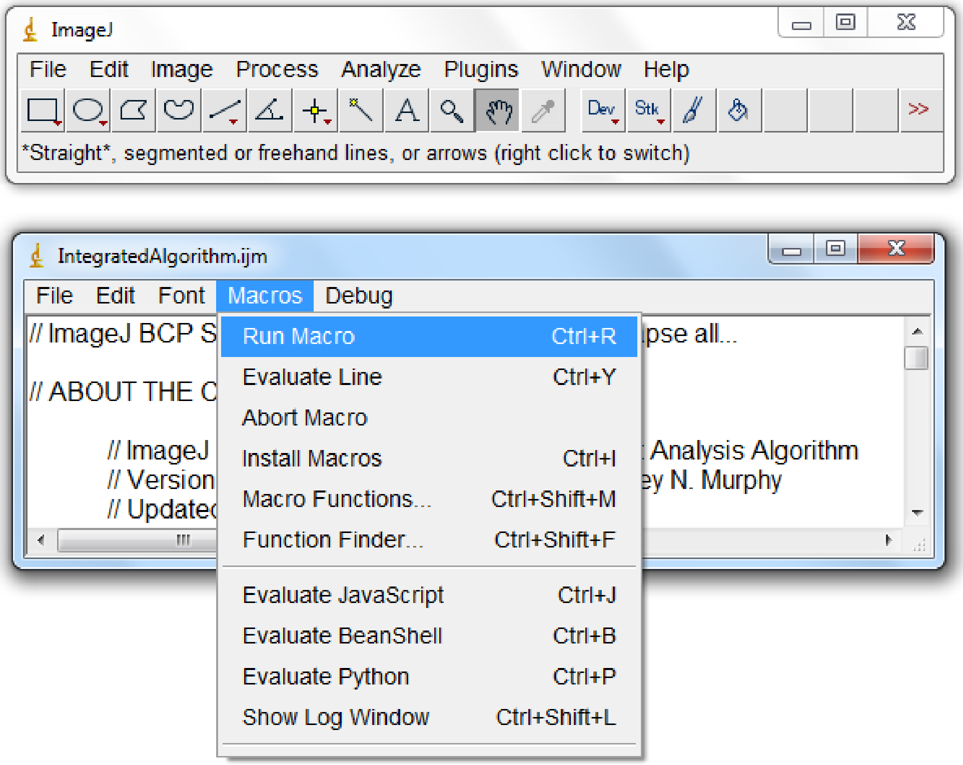

Screenshot: *Macro > Run Macro*

Input Required to Run the Algorithm:

6. Provide user inputs.

6A. Single or Multi Mode

You can choose either "**Single**" or "**Multi**".

- **Single** requires that an image is open and "active" (i.e. the last image selected prior to running the macro file). Any type of image can be used in this regard.
- **Multi** requires the selection of a directory containing either ***.TIF** or ***.PNG** files (this can be modified in the code). All files matching the extensions will be analyzed sequentially.
- **Batch Mode**, when checked, will hide all of the images and intermediate steps. A slight speed advantage is gained by not displaying the images.

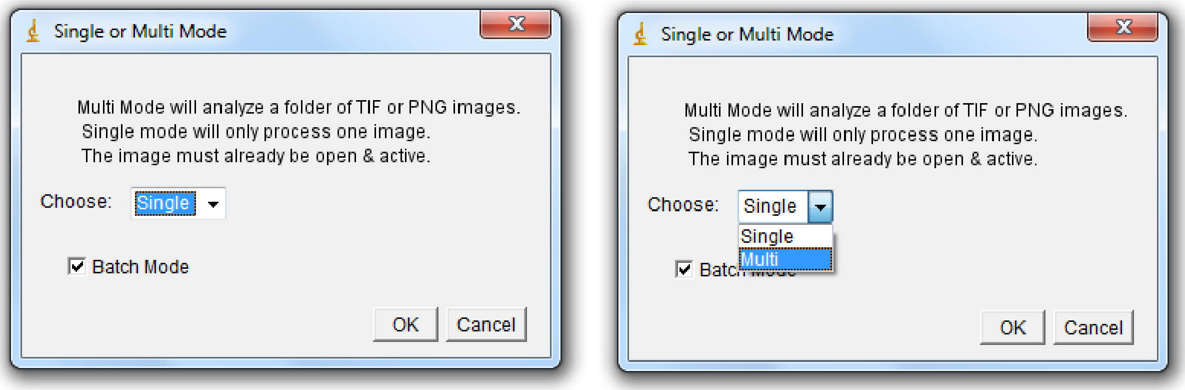

Screenshot: Choose either "**Single**" or "**Multi**".

6B. Determine Image Resolution

You must input how the image resolution will be determined. The method will be applied to all images being processed. The options are **Embedded**, **Specified**, or Special (**NINT_s4800**).

- **Embedded** uses whatever resolution and units are set in the image file. This will only work if the unit of resolution is set in "**um**", "**µm**", or "**nm**" (this can be modified in the code).
- **Specified** requires that all of the images possess a uniform resolution, which the user will set, using the two inputs:
  - Enter the dimensions of a pixel in the selected units:**1 pixel equals: ____ units**
  - Select the unit: **Unit: nm / µm**
  - Note that all pixels are presumed to have equal width and height
- **NINT_s4800** (**Special**) is used for images collected with the Hitachi S-4800 SEM. It's designed to determine the image resolution directly from the rendered scale bar.

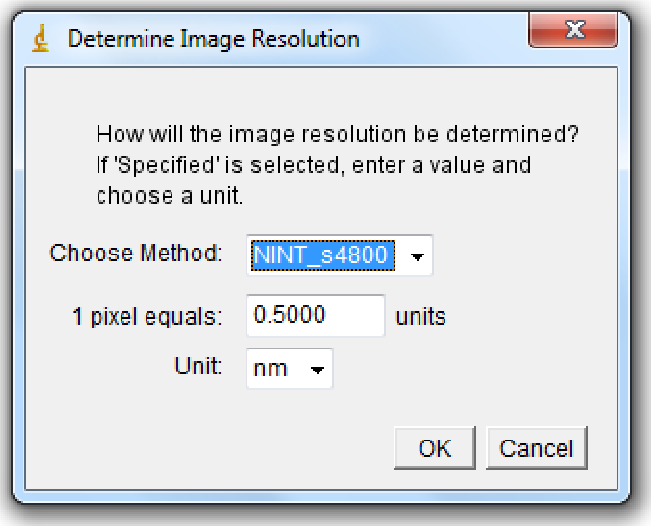

Screenshot: Determine Image Resolution

6C. Determine Line Period & Width

Input the method by which the period of the lines will be measured for use in subsequent parameters:

- **WLS** uses weighted least squares from particle analysis data to determine the period.
- **FFT** uses azimuthal (radial) the Fast Fourier
- **Specified** has the user input a specific **Period** in whatever **Units** (**pixels** / **nm** / **µm**)
- **Automatic** is the default method and uses a combination of the WLS and FFT options.

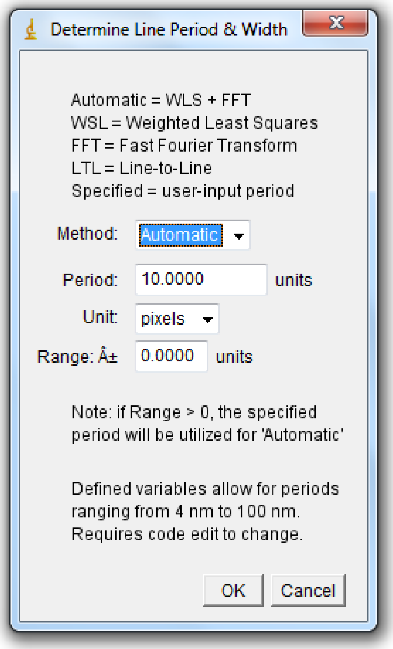

Screenshot: Determine Line Period and Width

6D. Additional Smoothing Procedures

Smoothing may be applied to the images prior to using the algorithm, if desired. This step provides for automatic smoothing of the image following determination of the period using FFT. The options are simple:

- **Automatic**: This is currently set to smooth the image using a median filter with a radius equivalent to 15% of the period.
- **None**: No additional smoothing will be applied. This is best if smoothing has already been applied or if the image is binary.
- **Median**: The image will be smoothed using a median filter with the set radius. Typically this gives superior results to Gaussian smoothing.
- **Gaussian**: The image will be smoothed using a median filter with the set radius.

Both Median and Gaussian use the other 3 inputs:

- **Radius __ pixels**: This sets the smoothing radius in pixels.
- **Relative**: If checked, this overrides “Radius __ pixels”, to use “% of period” instead.
- **Radius __ % of period**: If “Relative” is checked, this will set the smoothing radius relative to the period, as a percentage (out of 100) relative to the period. e.g. if “20%” was chosen, and the period was 30 pixels, the smoothing radius would be 6 pixels.

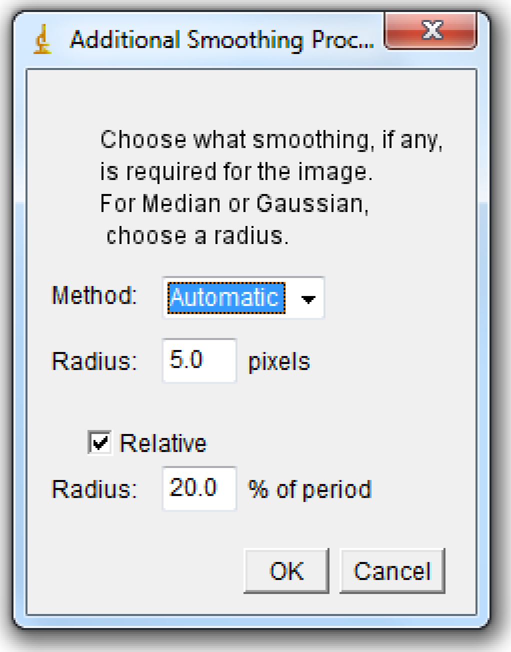

Screenshot: Additional Smoothing Procedures

6E. Choose Thresholding Method

Unless the image is already binary, the image requires thresholding. Several methods are available: some are included which may not be applicable. Typically, we either use the Built-in or the Auto-Local versions of the Otsu method. The Method Types available are:

- **Custom**: This currently uses the "Default dark" threshold, although it is essentially a reserved space for implementing custom or alternative thresholding methods.
- **Built-in**: These are universal (applying to the whole image) thresholding techniques built-in as defaults with ImageJ. Each method subdivides the image using a different statistical method basis. The
  - **Methods** available include: Default, **Huang, Intermodes , IsoData, IJ_IsoData , Li, Mean, Minimum , Otsu , & Triangle**.
- **Auto-Local**: These methods apply similar statistical methods, but in a regional fashion, using a radius around each point to determine the histogram of neighbours to each given point. Using a larger radius will require more time for the threshold to complete. Two additional input parameters are available, based on which **Auto-Local Type** is used:
  - None of the parameters: **Otsu**
  - Parameter 1: **Bernsen, Mean, Median, MidGrey**
  - Parameters 1 & 2: **Niblack, Phansalkar, Sauvola**
  - Additional details can be found at: http://fiji.sc/Auto_Local_Threshold
- **Radius**: This defines the area over which the local threshold is being performed. The unit for this, at present, is in pixels.

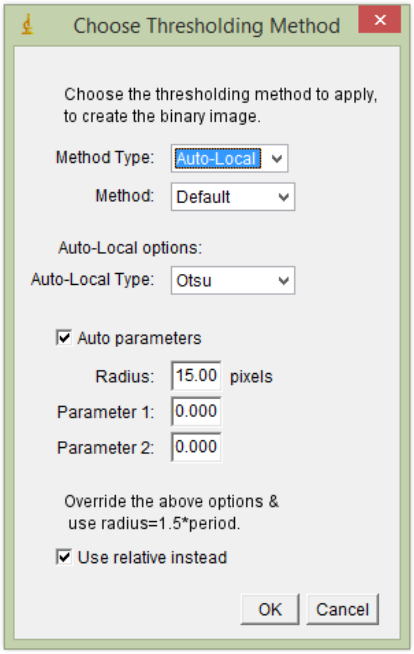

Screenshot: Choose thresholding method. “Use relative instead” will override absolute pixel units, and set the auto local threshold radius to 1.5 ✕ period (as determined by FFT).

6F. Crop Options

This is to automatically crop the image, as to remove edge effects, if necessary.

- **Crop**: **No** / **Yes**. Turns it on or off.
- **When**: **Stage 1** (before period estimate and smoothing) or **Stage 2** (after smoothing)
- **% or px**: **Percent** determines the amount cropped as a % (out of 100) of the original image; **Pixels** crops a specified amount from the edges.
- **Uniform** or **Non-Uniform**: If uniform is selected, the crop will be centred; if non-uniform is selected, an additional dialogue window will appear to enable for non-centred cropping.
- **Crop each edge**: The % or number of pixels to be cropped from each edge of the image.

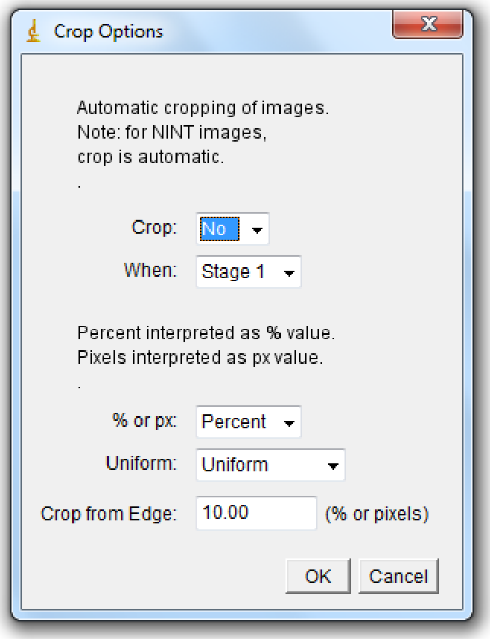

Screenshot: Crop Options

6G. Additional Analyses

An option which permits the user to save time if they do not desire to do the orientational domain map image, as shown in Figure 11. This can double the total amount of time required. The correlation data is retained however.

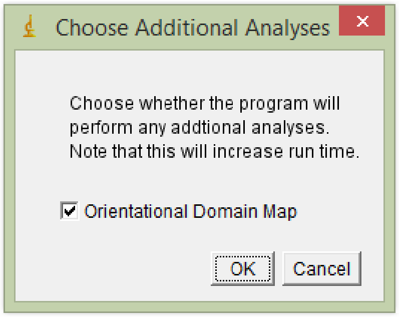

Screenshot: Choose Additional Analyses. Leaving the box unchecked skips that step.

6G. Choose a Folder (in Multi Mode)

Select the folder in which images to be analyzed are present. The method does not recursively search subfolders; only images in the immediate directory will be processed.

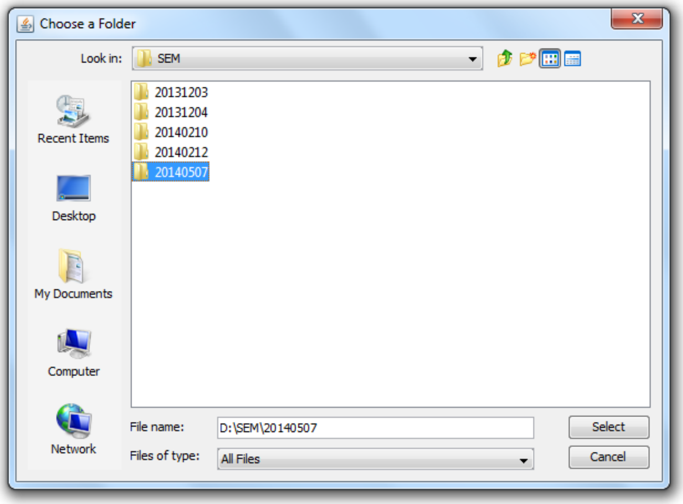

Screenshot: (Multi Mode) Choose a folder containing images to analyze.

6H. Choose save location for Output folder.

Select a location where the ***Output*** folder will be created. All files produced in the course of the analysis will be saved here. In Multi Mode, the output folder will contain a series of subfolders, 0, 1, 2, 3, etc… containing the files for each image processed, in sequence.

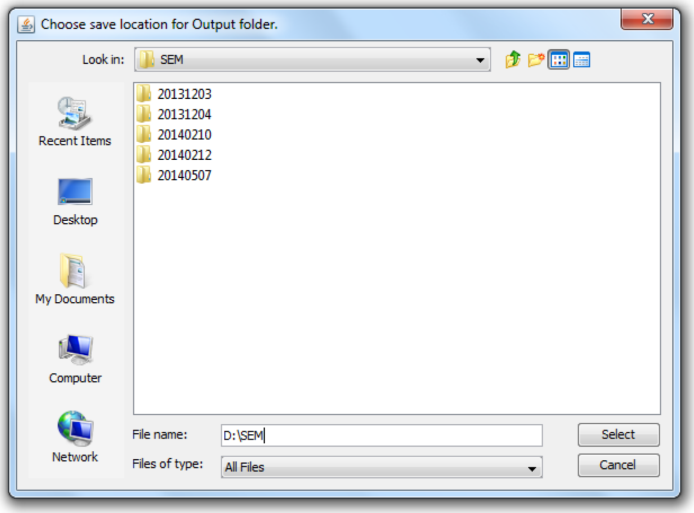

Screenshot: Choose save location for Output folder.

Sorting Through The Data

7. List of output files:

The program creates a directory within the "Save Location", which contains 1 folder for each image processed, each named with a sequential number, from 0 to N-1, where N is the total number of images processed. For example, the 6th image of a batch processed on 2015-01-31, starting at 12:15:30 :

C:/.../Save Location/Output_20150131_121530/5/

In the parent folder, there will be an accompanying file containing the filename of each image processed: Output_20150131_121530.txt; the general form is Output_YYYYMMDD_hhmmss.txt

Inside each image folder, there is a series of files produced. As the files do take up substantial space, saving can be turned off, should they be considered unnecessary. The list of files currently output is as follows:

Images

- [image000.tif] The original image
- [image001.tif] After cropping (for scalebar or other reasons)
- [image002.tif] After smoothing
- [image003.tif] After smoothing (if additional)
- [image004.tif] Binary image (black and white)
- [image_positive_broken.png] Binary image with junctions removed
- [image_broken_marked.png] Binary image with junctions removed
- [image_broken_skeleton.png] Skeleton image with junctions removed
- [correlation_data_skeleton_1.png] Skeleton image used for generating orientation data
- [image_positive_lines.png] Binary of positive lines only
- [image_negative_lines.png] Binary of negative lines only
- [image_positive_skeleton] Binary skeleton of positive lines only
- [image_negative_skeleton.png] Binary skeleton of negative lines only
- [image_positive_dots.png] Binary of positive dots only
- [image_negative_dots.png] Binary of negative dots only
- [positive_j_skeleton.png] Skeleton of positive lines with junctions and terminal points marked
- [negative_j_skeleton.png] Skeleton of negative lines with junctions and terminal points marked
- [image_figure_4_b.tif/png] Defect features in the positive phase only
- [image_figure_4_c.tif/png] Defect features in the negative phase only
- [image_validation_defects.png] Skeletons of both phases, with junction and terminal point pixels coloured for identification; Junctions with >3 branches are identified with a circle
- [image_regions_defects.png] Junction and terminal point features all marked by simple yellow-green circles; Dot features are coloured in magenta (pos) or teal (neg)
- [image_regions_defects_NEW.png] All defects marked and identified, according to legend shown in Figure S12 below

- [DomainMap.png/tif] Image showing the orientation of lines. (Optional)

Graphs

- [correlation_data_CL_0.png] Graphical data of the correlation analysis.

Data Files

- [correlation_data_CL_0.xls] Correlation data values for graphing
- [d_mode_FFT.xls] Fast Fourier transform values for diagnostic check
- [defect_coordinates.xls] List of defect coordinates
- [feature_particle_analysis.xls] Details of the particle analysis
- [feature_particle_analysis_1.xls] Details of the particle analysis
- [feature_particle_analysis_2.xls] Details of the particle analysis
- [line_roughness_mesurements.xls] Line roughness measurements for each line segment
- [outputTD_horizontal.xls] Output of primary data; arranged in two rows
- [outputTD_vertical.xls] Output of primary data; arranged in two columns

Log Files

- [Output_Log.txt] Log of data and events during image processing

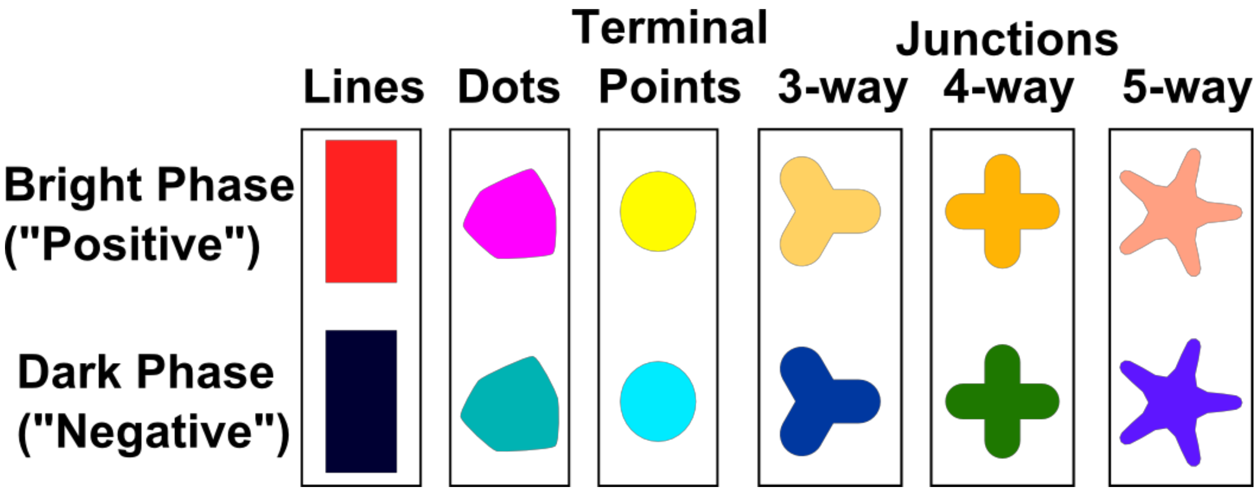

Figure S12. Legend used for depicting defects and line features in confirmation images.

8. Summarizing data:

- A Python script for extracting data and summarizing the output from the “outputTD” files for multiple runs is available with the code on GitHub.
- An additional Python script to assist with plotting and exploratory data analysis is also available with the code on GitHub.

Modifying the Code

9. ImageJ Documentation: Details of the built-in functions of the ImageJ macro language can be found at:

- http://rsb.info.nih.gov/ij/developer/macro/macros.html
- http://rsbweb.nih.gov/ij/developer/macro/functions.html

The macro can be modified and interested persons are welcome to contact the authors for additional details.

10. Editing Macro Files: Although ***.ijm** files can be opened and edited with any text editor, a syntax highlighter for **jEdit** is available, and can be used as a plugin with ImageJ:

- jEdit: http://www.jedit.org
- Syntax Files: http://imagejdocu.tudor.lu/doku.php?id=plugin:utilities:ij_ed:start (created by Jerome Mutterer)
